# Target Metabolome Profiling-Based Machine Learning as a Diagnostic Approach for Cardiovascular Diseases in Adults

**DOI:** 10.3390/metabo12121185

**Published:** 2022-11-27

**Authors:** Natalia E. Moskaleva, Ksenia M. Shestakova, Alexey V. Kukharenko, Pavel A. Markin, Maria V. Kozhevnikova, Ekaterina O. Korobkova, Alex Brito, Sabina N. Baskhanova, Natalia V. Mesonzhnik, Yuri N. Belenkov, Natalia V. Pyatigorskaya, Elena Tobolkina, Serge Rudaz, Svetlana A. Appolonova

**Affiliations:** 1 World-Class Research Center Digital Biodesign and Personalized Healthcare, I.M. Sechenov First Moscow State Medical University, 119435 Moscow, Russia; 2 Laboratory of Pharmacokinetics and Metabolomic Analysis, Institute of Translational Medicine and Biotechnology, I.M. Sechenov First Moscow Medical University, 119435 Moscow, Russia; 3 Hospital Therapy N°1 Department of the N.V. Sklifosovsky Institute of Clinical Medicine, I.M. Sechenov First Moscow Medical University, 119992 Moscow, Russia; 4 Department of Industrial Pharmacy, Institute of Vocational Education I.M. Sechenov First Moscow State Medical University, 119435 Moscow, Russia; 5 Institute of Pharmaceutical Sciences of Western Switzerland, University of Geneva, 1206 Geneva, Switzerland

**Keywords:** metabolites, amino acids, acylcarnitines, tryptophan catabolism, methylarginines, cardiovascular disorders, hypertension, coronary heart disease, translational medicine, machine learning

## Abstract

Metabolomics is a promising technology for the application of translational medicine to cardiovascular risk. Here, we applied a liquid chromatography/tandem mass spectrometry approach to explore the associations between plasma concentrations of amino acids, methylarginines, acylcarnitines, and tryptophan catabolism metabolites and cardiometabolic risk factors in patients diagnosed with arterial hypertension (HTA) (n = 61), coronary artery disease (CAD) (n = 48), and non-cardiovascular disease (CVD) individuals (n = 27). In total, almost all significantly different acylcarnitines, amino acids, methylarginines, and intermediates of the kynurenic and indolic tryptophan conversion pathways presented increased (*p* < 0.05) in concentration levels during the progression of CVD, indicating an association of inflammation, mitochondrial imbalance, and oxidative stress with early stages of CVD. Additionally, the random forest algorithm was found to have the highest prediction power in multiclass and binary classification patients with CAD, HTA, and non-CVD individuals and globally between CVD and non-CVD individuals (accuracy equal to 0.80 and 0.91, respectively). Thus, the present study provided a complex approach for the risk stratification of patients with CAD, patients with HTA, and non-CVD individuals using targeted metabolomics profiling.

## 1. Introduction

Cardiovascular diseases (CVDs) are the leading cause of death globally and are responsible for approximately 18 million deaths each year [1]. Conditions such as hypertension, hypercholesterolemia, and type 2 diabetes mellitus are well known as risk factors for cardiovascular diseases [2]. Cardiometabolic alterations occur in association with asymptomatic metabolic perturbations [3,4]. These include, for example, an imbalance in general metabolism, signaling pathways, or alterations in macronutrient biosynthesis and degradation. Improving the understanding of these metabolic changes and identifying biomarkers that reflect these alterations may provide a deeper understanding of the underlying pathophysiological processes and for translation of this knowledge to elucidate early-stage development of CVDs, especially when standard clinical methods are limited because these alterations occur at subclinical, asymptomatic levels [5]. Omics technologies such as metabolomics profiling offer an opportunity to recognize subtle alterations in the metabolome that occur before the appearance of phenotypic changes common for the disease [6]. Plasma is one of the most common biofluids typically utilized in metabolomics profiling due to its stability and the relatively noninvasive sampling techniques involved [7,8,9].

Characterization of differences among altered metabolic pathways may provide insights into the pathology, prognosis, and early diagnosis of CVDs [10]. The level of understanding of metabolic alterations occurring in organs and tissues associated with the development of cardiovascular pathologies is relatively high. At present, multiple studies have connected alterations in the concentration levels of cholesterol and triglycerides, phosphatidylcholines, and several amino acids, including branched-chain amino acids, unsaturated lipids, choline, and trimethylamine-N-oxide, with CVD [11,12,13,14,15].

However, there is no clarity on the most highly specific and sensitive biomarkers for early coronary artery disease (CAD) diagnosis. Moreover, the prediction of the progression and adverse outcomes in asymptomatic stages of CAD are unclear. The cardiovascular disease continuum (CVDC) is a sequence of cardiovascular events that start from a cluster of cardiometabolic risk factors, including dyslipidemia, smoking, and excessive visceral adiposity, among others. This pathogenic process, if not treated properly at an early stage, often progresses into atherosclerosis, myocardial remodeling, increased vascular pressure, cardiac overload, myocardial wall remodeling, heart dilation, and heart failure [16]. Early elucidation of metabolic alterations underlying metabolic syndrome, arterial hypertension (HTA) and CAD are crucial for the prevention of further cardiovascular complications. It has been described that both HTA and CAD are characterized by endothelial dysfunction, instability of atherosclerotic plaques, and an increase in myocardial oxygen demand [17]. Moreover, application of metabolomics profiling from the view of personalized medicine may support the accurate measurement of the patient’s phenotypes [18] as well as the further introduction of the obtained information into clinical practice.

From the view of metabolomics studies, it has been reported that cardiometabolic alterations are accompanied by changes in the concentration levels of several plasma amino acids [19,20], acylcarnitines [21], and biodegradation products related to tryptophan metabolism [22]. However, the mechanisms and diagnostic biomarkers at different stages of CVDC remain unclear.

Prediction of the clinical outcome based on metabolomics profiles is a relatively complicated task due to the complexity and nonlinear representation of the data. Artificial intelligence tools based on supervised machine learning (ML) classification algorithms may serve for the preliminary prediction of CVD as well as its progression. Based on the developed ML-derived models, it becomes possible to perform quantitative predictions as well as to identify meaningful potential biomarkers through the selection of informative features, facilitating preliminary hypothesis-driven research.

Therefore, the application of metabolomics profiling with the aim of distinguishing patients at different stages of CVDC represents an opportunity for translational medicine to systematically identify potential early clinical markers of CVD. The goal of the present study was to explore the association between plasma concentrations of amino acids, methylarginines, acylcarnitines, and tryptophan catabolism metabolism with cardiometabolic risk factors in adults diagnosed with HTA and CAD compared to non-CVD individuals.

## 2. Materials and Methods

### 2.1. Study Design and Participants

This cross-sectional study was conducted in 136 adults. The main group comprised 109 patients diagnosed with CVD (CVD group): 61 had HTA (HTA subgroup), and 48 were patients with CAD (CAD subgroup). A non-CVD group comprised 27 participants without clinical and laboratory signs of CVD. All participants were recruited from Cardiology Department No. 1, Clinical Hospital No. 1 at Sechenov University, Moscow, Russia, between 2018 and 2020. Adults with hypertension were patients diagnosed with systolic blood pressure (SBP) above 140 mmHg and/or diastolic blood pressure (DBP) above 90 mmHg. Patients were defined as those with CAD in case of stress-induced myocardial ischemia, available stenosis of the coronary arteries (CAs) which was diagnosed using coronary angiography or computed tomography (CT) or with history of myocardial infarction. The non-CVD group comprised 27 adults without clinical and laboratory signs of CVD (the absence of cardiovascular pathologies including HTA, CAD, congenital and acquired heart defects, left ventricular hypertrophy, cardiomyopathy, infiltrative heart disease, heart failure, rhythm disturbances (atrial fibrillation/flutter, ventricular arrhythmias), and cardiac conduction disorders. Ten of them were defined as healthy, and seventeen of them had metabolic disorders (body mass index (BMI) ≥ 25 kg/; dyslipidemia). All patients were recommended a balanced diet. Acetaminophen, all vitamins, minerals, amino acids, and dietary supplements, including sports drinks and energy drinks, were excluded 4 days before blood sampling. Creatinine, alpha-ketoglutarate, supplements containing malic acid, as well as a salt of citric acid, maleic, or any salts of orotic acid were stopped 4 days before blood sampling. Sweeteners (aspartate, among others) and monosodium glutamate were excluded 24 h before inclusion.

### 2.2. Exclusion Criteria

Secondary hypertension, cerebrovascular disorders (dementia, at least 6 months apart from stroke), acute and chronic kidney failure, chronic pulmonary heart disease, signs and symptoms of liver disease (without cytolysis syndrome and liver failure), portal hypertension, asthma, chronic obstructive pulmonary disease, gastric or duodenal ulcer in the acute phase, chronic pancreatitis in the acute phase, malignant neoplasms, thyroid diseases (hypothyroidism and hyperthyroidism), Cushing syndrome, type 1 diabetes, type 2 diabetes mellitus decompensation, thrombocytopenia, hemorrhagic syndrome, autoimmune diseases, mental illness or disability, alcoholism, drug addiction, substance abuse, pregnancy, and breast-feeding were the exclusion criteria. The non-CVD group included patients without hypertension, CD, congenital and acquired heart defects, left ventricular hypertrophy, cardiomyopathy, infiltrative heart diseases, heart failure, rhythm disorders (atrial fibrillation/flutter, ventricular arrhythmias), and cardiac conduction, in addition to the abovementioned disorders. Anthropometric measurements, complete blood count and biochemical analysis, electrocardiography (ECG), echocardiography (Echo), renal ultrasonography and carotid ultrasound investigation, 24 h ambulatory blood pressure monitoring, 24 h ECG monitoring, and metabolomics profiling were measured in all participants.

### 2.3. Ethical Considerations

All experiments were approved by the Ethics Committee of I. M Sechenov First Moscow State Medical University, Moscow, Russia (Document #05-17, April 2017) in conformity with the ethical principles for medical research involving humans stated in the Declaration of Helsinki. Written informed consent was signed by all the participants before the beginning of the study.

### 2.4. Anthropometric Measurements

Weight was measured to the nearest 0.1 kg using an electronic scale (Seca Ltd., Hamburg, Germany). Height was measured using a stadiometer to the nearest 0.1 cm (Seca Ltd., Hamburg, Germany). Body mass index (BMI) was computed using the following formula: (weight in kg divided by height in meters)^2^ to classify nutritional status as underweight or normal (BMI ≤ 25 kg/m^2^), overweight (BMI = 25–30 kg/m^2^), or obese (BMI ≥ 30 kg/m^2^) [23].

### 2.5. Echocardiographic Examination

A two-dimensional echocardiography technique on modes M and B and using pulse-wave and continuous-wave Doppler in the supine position using a Vivid7 Dimension/Vivid 7 PRO echocardiograph version 6.0.x, Germany, was performed. Abnormal posterior and septal wall thickness were defined as <10 mm [24], and abnormal left ventricular ejection fraction (LVEF) was defined as <50% [24]. Abnormal diastolic function was defined as a mitral E/A ratio ≤0.8 to ≥2 [22]. Twenty-four-hour ambulatory blood pressure assessment monitoring SBP and DBP were determined in twenty-four-hour cycles (day/night) using Spacelabs Medical portable equipment (Nuremberg, Germany) [25].

### 2.6. Smoking and Daily Drug Use

Smoking categorized as “yes” or “no” at the time of the medical evaluation was defined as “yes” if smoking at least one cigarette per day. The daily use of drugs was defined as yes or no based on the daily self-reported use of angiotensin-converting enzyme (ACE) inhibitors, statins, and beta-blockers.

### 2.7. Biochemical Analyses

Blood samples were collected using ethylenediaminetetraacetic acid (EDTA) tubes after overnight fasting. Samples were centrifuged (2000 rpm, 4 °C) for 20 min to separate plasma and were stored at −80 °C. Blood samples were shipped to the Interclinical Biochemical Laboratory at Sechenov University for biochemical analyses, including the measurements of circulating glucose, creatinine, uric acid, total cholesterol (TC), high-density lipoprotein cholesterol (HDL-C), low-density lipoprotein cholesterol (LDL-C), plasma triglycerides (TGs), and thyroid hormones. Elevated glucose concentrations were categorized as >100 mg/dL [26]. Total cholesterol, LDL-C, VLDL-C, and TG were abnormal if >200 mg/dL, >100 mg/dL, >30 mg/dL, and >150 mg/dL, respectively [27]. Abnormal HDL-C was defined as <40 mg/dL in men and <50 mg/dL in women [27]. Abnormal serum creatinine was defined as >110 μmol/L for men and <45 or >90 μmol/L for women [28]. Abnormal uric acid was defined as >6.99 mg/dL and >5.6 mg/L in men and women, respectively [27]. Glucose, serum lipids, creatinine and uric acid were determined using an ABX Pentra 400 analyzer (Horiba ABX SAS, Montpellier, France). Extra plasma aliquots were shipped to the Laboratory of Pharmacokinetics and Metabolomics Analysis, Institute of Translational Medicine and Biotechnology at Sechenov University for metabolomics profiling.

### 2.8. Chemicals and Reagents

Standard solutions for acylcarnitine and amino acid profiling, methanol, formic acid, bovine serum albumin (BSA), sodium chloride, 6-hydroxynicotinic acid, 3-indole acrylic acid, neopterin, biopterin, l-tryptophan, and ascorbic acid were received from Sigma–Aldrich (USA). Acetonitrile was obtained from Chromasolv^®^ (Sigma-Aldrich Chemie GmbH, Buchs, Switzerland). Ultra-pure water was obtained from a Millipore Milli-Q water purification system (Millipore Corporation, Billerica, MA, USA). Isotope-labeled standard solutions of metabolites related to tryptophan catabolism were received from Toronto Research Chemicals (Toronto, ON, Canada). Isotope-labeled standard solutions for acylcarnitine and amino acid profiling were purchased from MassChrom Amino Acids and Acylcarnitines Non-Derivatised 57,000 Kit (Chromsystems, Germany).

### 2.9. Amino Acid Determination

A quantification method for 19 amino acids was developed. Stock amino acid solutions were prepared in a 100 mM 10% methanol–water solution. Preparation of working solutions was performed by serial dilution of standards with 10% methanol in water. Calibration solutions and QC samples were prepared from a surrogate matrix consisting of 2% bovine serum albumin in PBS buffer. The sample preparation procedure was as follows: a 10 μL aliquot of each plasma sample (calibrator or QC sample) was mixed with 50 μL of ISTD mix solution and 40 μL of methanol for protein precipitation. Following 10 min of incubation, the samples were centrifuged for 5 min at 13,000× *g*. After that, 40 μL of the received supernatant was transferred into an LC–MS vial and diluted with 40 μL of water for the subsequent LC–MS/MS analysis.

Instrumental analysis was performed using a Waters Acquity I high-performance liquid chromatography (HPLC) system coupled to a Waters TQ-S-micro triple quadruple mass spectrometer (Waters Corp, Milford, CT, USA). Chromatographic separation was achieved using a Waters ACQUITY BEH C18 column 1.7 μm, 100 mm × 2.1 mm (Waters, USA). The mobile phases consisted of LC–MS-grade water with 0.1% formic acid (phase A) and acetonitrile containing 0.1% formic acid (phase B) with a flow rate set at 0.3 mL/min. The gradient program was as follows: 1 min: 1% B; 3 min: 20% B; 5 min: 90% B; 8 min: 90% B; 8.1 min: 1% B; 12 min: 1% B. The column temperature was maintained at 40 °C. Ionization was performed using electrospray ionization in positive mode. The determination of ions was achieved in multiple reaction monitoring (MRM) mode (Supplementary Table S1). Mass spectrometric conditions were as follows: dwell time: 0.019–0.025 s; capillary voltage: 1 kV; collision gas medium: nitrogen; source temperature: 150 °C. Preprocessing and data import were performed in TargetLynx software (Waters, Milford, MA, USA).

Based on the received quantitative results, the GABR ([Arginine]/([Ornithine] + [Citrulline])) ratio, AOR ratio ([Arginine]/[Ornithine]) and Fisher ratio (([Leucine] + [Isoleucine] + [Valine])/([Tyrosine] + [Phenylalanine])) were calculated.

### 2.10. Asymmetric Dimethylarginine and Symmetric Dimethylarginine Quantification

Sample preparation for asymmetric dimethylarginine (ADMA) and symmetric dimethylarginine (SDMA) determination was performed as follows: a 100 μL aliquot of each plasma sample (calibrator or QC sample) was mixed with 50 μL of isotopically labeled internal standards (ISTD) solution (D7-Arg, 1.55 μM) and 40 μL of methanol in a microtiter plate for protein precipitation. After 10 min of incubation, the microtiter plate was centrifuged for 5 min at 13,000× *g*. Then, 40 μL of supernatant was transferred into a vial and mixed with 40 μL of water, and 1 μL of the received solution was injected into the LC–MS/MS system.

Samples were analyzed using a Waters Acquity I HPLC system coupled to a Waters TQ-S-micro triple quadruple mass spectrometer (Waters Corp, Milford, USA). Chromatographic separation was conducted using a Waters ACQUITY BEH C18 column 1.7 μm, 100 mm × 2.1 mm (Waters, USA). The mobile phases consisted of water with 2 mM ammonium formate and 0.015% heptafluorobutyric acid (mobile phase A) and methanol containing 2 mM ammonium formate and 0.015% heptafluorobutyric acid (phase B). The elution program was as follows: 1% B at 1 min, 20% B at 5 min, 90% B at 6 min, 90% B at 11 min, 1% B at 11.1 min, and 1% B at 15 min. The flow rate and column temperature were 0.3 mL/min and 40 °C, respectively. Mass spectrometric detection was achieved in MRM mode (Supplementary Table S2) with a dwell time of 20 ms. Capillary and cone voltages were 1 and 19 V, respectively. The source temperature was set at 150 °C, the desolvation temperature was set at 500 °C, and the source and desolvation gas flow rates were 10 L/min and 1000 L/h, respectively.

### 2.11. Acylcarnitine Determination

Forty-four acylcarnitines were quantified using the following method. Sample preparation was performed under the following conditions: 10 µL of each sample (calibrator or QC) was mixed with 50 µL of ISTD solution and 40 µL of acetonitrile. The mixture was vortexed and centrifuged at 13,000 rpm for 10 min. Furthermore, 80 µL of the received supernatant was mixed with the same volume of water, and the resulting mixture was ready for the subsequent UPLC–MS/MS analysis. A Waters Acquity I HPLC system coupled to a Waters TQ-S-micro triple quadruple mass spectrometer (Waters Corp, Milford, CT, USA) was used. Chromatographic separation was conducted using a Waters ACQUITY BEH C18 column 1.7 μm, 100 mm × 2.1 mm (Waters, USA). Both mobile phase A (water) and phase B (acetonitrile) contained 0.1% formic acid. The linear gradients were as follows: 1% B at 1 min, 20% B at 3 min, 90% B at 5 min, 90% B at 8 min, 1% B at 8.1 min, and 1% B at 12 min. The flow rate was 0.3 mL/min, and the column temperature was set at 40 °C. The injection volume and flow rate were 1 µL. MS detection was achieved by multiple reaction monitoring (MRM) (Supplementary Table S3) with a dwell time of 20 ms. Capillary and cone voltages were 1 and 19 V, respectively. The source temperature was set at 150 °C, the desolvation temperature was set at 500 °C, and the source and desolvation gas flow rates were 10 L/min and 1000 L/h, respectively. Metabolite concentrations were calculated according to the signal intensity of analytes and appropriate internal standards.

### 2.12. Tryptophan Catabolism Metabolite Determination

The profiling of metabolites related to tryptophan catabolism included the determination of 20 analytes. Sample preparation was performed as follows: 100 µL of plasma (calibrators or QCs) was mixed with the internal standard solution (10 µL of stock solution of 10 µg/mL solution of 2-hydroxynicotinic acid) and 400 µL of acetonitrile. Subsequently, the mixture was vortexed, centrifuged for 10 min at 13,000 rpm and evaporated to dryness in a vacuum centrifuge evaporator at 37 °C. The residues were further reconstituted with 100 µL of a solution of 0.02% ascorbic acid in 10% methanol, centrifuged, and transferred into an LC–MS vial. Five microliters of the extract was injected into the liquid chromatograph for the subsequent LC–MS/MS analysis.

Instrumental analysis was performed using an Agilent 1200 liquid chromatograph coupled to a 6450C tandem mass spectrometer (Agilent Technologies, Paolo Alto, CA, USA). The chromatographic separation was achieved using a Discovery PFP HS F5 2.1 × 150, 3 µm column (Supelco Inc, St. Louis, Missouri, USA) equipped with a Waters WAT084560 guard column (Waters Inc. USA). The column temperature and the flow rate were set at 40 °C and 0.4 mL/min, respectively. The mobile phases consisted of 0.1% formic acid aqueous solution (phase A) and acetonitrile (phase B). The gradient program was as follows: 0 min: 1% B; 4 min: 10% B; 9 min: 90% B; 10 min: 90% B; 10.1: 1% B; 12 min: 1% B. Electrospray ionization was operated in positive mode. The main MS parameters were as follows: gas temperature: 300 °C; gas flow: 8 L/min; nebulizer gas: 20 psi; sheath gas heater: 300; sheath gas flow rate: 10 L/min; capillary voltage: 3500 kV. Analytes were detected using the MRM transitions presented in Supplementary Table S4.

### 2.13. Validation of the Methods

The presented methods were validated in accordance with the US FDA and EMA guidelines for bioanalytical method validation (EMA, 2019; USFDA, 2018). Validation included assessment of selectivity, linearity, precision and accuracy, recovery, matrix effect, and stability of the method. Quality control samples were used for monitoring of the data enrichment and instrumental performance. The calibration characteristics of the analytes were determined based on the analysis of eight calibrators in three replicates examined through three analytical runs. Calibration curves were fitted using a weighted linear regression model. Inter- and intra-assay precision and accuracy were assessed using QC samples in six replicates through three analytical runs. To assess the stability of the methods, QC samples at low QC (LQC) and high QC (HQC) levels were utilized. Stability was assessed in working solutions of the analytes that were stored at room temperature (21 ± 3 °C); in biological samples stored in an autosampler for 24 h at 10 ± 0.5 °C; and in biological samples stored at 35 ± 1 °C for 20 days. Recovery and matrix effects were assessed in QC samples at low and high concentration levels. Inter- and intrabatch precision and accuracy were assessed using QC samples in six replicates through three analytical runs. The relative standard deviation of all tested QC samples was within 15%. The calibration curves were linear. The lower limit of quantification for all analytes was 1 μM. The inter- and intrabatch accuracy and precision for all of the analytes were below 5.4% and 7.1%, respectively. The matrix effect ranged from 95.1% to 99.4%, and the recovery was between 93.8 and 99.1%. Sample preparation for each profiling method was performed simultaneously.

### 2.14. Statistical Analysis

Statistical analysis was performed using the Stats package in Python software. The distribution of the variables was checked with the Shapiro–Wilk test. Further analysis of variance was performed using parametric ANOVA or the nonparametric Kruskal–Wallis test (*p* value < 0.05). The correction of the *p* value was performed using the Benjamini–Hochberg false discovery rate (BH-FDR) method, providing q values. Identification of significant features was performed through the analysis of the whole data matrix, where all values lower than 0.05 were considered significant.

The diagnostic accuracy of the selected metabolites was tested through calculation of the areas under the curve (AUCs) obtained from receiver operating characteristic curve analyses by comparing the non-CVD group with the CVD group (combined HTA and CAD patients) and the non-CVD group with the HTA group. Heatmap correlation matrices were assessed with Spearman correlations using the Seaborn package (Python) to investigate the relationships between cardiometabolic risk factors and metabolomics profiling.

### 2.15. Machine Learning Methods

Machine learning (ML) methods provide an opportunity to process high-dimensional datasets. In the current study, the dependent variable was first considered multiple (patients with CAD, patients with HTA and non-CVD individuals), while further patients from the CVD groups were combined to build a binary classification model. Multiple classification was performed using four different algorithms: random forest, gradient boosting, multiple neural networks, and support vector machines. Binary classification was performed through the application of six machine learning methods, including logistic regression, random forest classifier, multiple neural network, gradient boosting, support vector classifier, and bagging classifier. Hyperparameter optimization of the developed models was performed using the sklearn GridSearchCV tool (sklearn, Python).

### 2.16. Logistic Regression

Logistic regression is one of the most widespread ML algorithms typically applied for binary classifications. It aims to predict the probability of the sample being referred to the presented groups according to the metabolite peak intensities by comparison with logistic curves. In the case of penalized logistic regression, the model applies a built-in stepwise variable selection process that serves to eliminate the powerless variables in the classification of the two groups. The maximum number of iterations in the fitted models was equal to 100, and the ‘newton-cg’ solver was used.

### 2.17. Random Forest Classifier

Random forest (RF) is an ML algorithm that utilizes a specific combination of tree predictors, where each tree is constructed independently based on the random set of observations. Thus, the distribution of the trees in the forest is the same, whereas each tree provides the best classification outcome. The final ‘score’ of the model is derived from the scaled summary of the tree’s outcomes. Optimization of the main hyper parameters of the random forest classifier resulted in the following hyper parameters: [‘bootstrap’: false; ‘maximum depth’: 120; ‘maximum features’: ‘sqrt’; minimum samples leaf’: 1; ‘minimum samples split’: 3; ‘n_estimators’: 100 for multiclass classification] and [‘bootstrap’: false; ‘maximum depth’: 3; ‘maximum features’: ‘sqrt’; minimum samples leaf’: 3; ‘minimum samples split’: 3; ‘n_estimators’: 200 for binary classification].

### 2.18. Multiple Neural Networks

The multiple neural network algorithm was released through the utilization of a multilayer perceptron classifier (MLPC). MLPC is based on the backpropagation algorithm that computes the gradient of the loss function based on the weights of the network of a single input–output case. The hyperparameters utilized for this algorithm in multiclass classification included ‘activation’: ‘relu’; ‘alpha’: 0.1; ‘max_iter’: 150; ‘solver’: ‘lbfgs’. The binary classification parameters were as follows: activation: ‘relu’; alpha: 1e-5; maximum iteration: 2000; solver: ‘sgd’.

### 2.19. Gradient Boosting

Unlike random forest, where trees are formed independently, a classification algorithm using gradient boosting is constructed such that each model compensates for errors of the previous model. In this case, target outcomes are generated on the basis of the error gradient in accordance with the prediction. To apply the gradient boosting classifier to the multiple classification problem, the following hyperparameters were tuned: maximum depth: 5; minimum samples leaf: 5; minimum samples split: 50; n_estimators: 15. At the same time, binary classification parameters were as follows: maximum depth: 13; maximum features: ‘auto’; minimum samples leaf: 1; minimum samples split: 4.

### 2.20. Support Vector Machine

The application of the support vector machine (SVM) algorithm for classification purposes is based on the principle of structural risk minimization that serves to identify the lowest-probability error. The SVM classification method is applicable for nonlinear classification issues by using a kernel function and is relatively insensitive to the high dimensionality of the data in comparison to several other ML methods [29]. The utilized algorithm for multiple classification was performed using the following hyperparameters: C = 2, decision_function_shape = ‘ovo’, probability = True.

### 2.21. Bagging Classifier

Bagging classification is a machine learning approach released as an ensemble of meta-estimators that train basic classifiers using random subsets of the original dataset. As a result, the final prediction is formed according to the predictions of each fitted subset. Therefore, the bagging classifier allows a decrease in the variance of the trained classifier through the application of randomization during its construction procedure. The bagging algorithm was used for classification of the data in this study through utilization of the following parameters: number of estimators, 15; maximum samples, 1; maximum features, 1.

To assess the power of different models aiming to determine the CVD state, the following metrics were applied: values of the confusion matrix, accuracy, AUC-score, Cohen’s Kappa, Matthew’s correlation coefficient, and log loss. Quality of the binary classification was assessed using values of the confusion matrix, accuracy, AUC-score, precision and recall, and F1-score. Confusion matrix values comprised TP (patients correctly diagnosed as having CVD); FP (non-CVD individuals incorrectly diagnosed as patients); TN (non-CVD individuals correctly found as non-CVD); FN (CVD patients incorrectly defined as non-CVD individuals).

## 3. Results

The present study aimed to assess the meaningful metabolites that could provide accurate separation of patients with HTA, patients with CAD, and non-CVD individuals. The analysis was performed using targeted metabolomics profiling of the following metabolic panels: amino acids, methylarginines, acylcarnitines, and tryptophan catabolism intermediates.

### 3.1. General Characteristics of the Participants

Nearly half of the participants in each group were female. It is worth noting that the CVD groups [62 (55–69) years in HTA and 65 (59–71) years in CAD] were older (*p* < 0.001) than the non-CVD group [48.5 (43–51) years]. Patients in the CVD groups had a higher BMI (*p* < 0.05) than those in the non-CVD group [31 (29–34) kg/m^2^ and 30 (26–34) kg/m^2^ in the HTA and CAD groups versus 26.5 (24.7–28.0) kg/m^2^ in the non-CVD group]. Posterior wall thickness, septal thickness, and E/A ratios were significantly altered (*p* < 0.001) when compared to the non-CVD group. Systolic blood pressure at day and night according to ambulatory blood pressure monitoring was higher in the HTA group than in the non-CVD group, as expected. Plasma glucose levels were significantly higher (*p* < 0.05) in the CVD groups than in the non-CVD group. It is important to note that 53.5%, 47.5%, and 51.5% of the patients belonging to the HTA and CAD groups were treated with ACE inhibitors, beta-blockers, and statins, respectively (Table 1).

### 3.2. Heatmap Correlation Matrices

**Figure 1:**
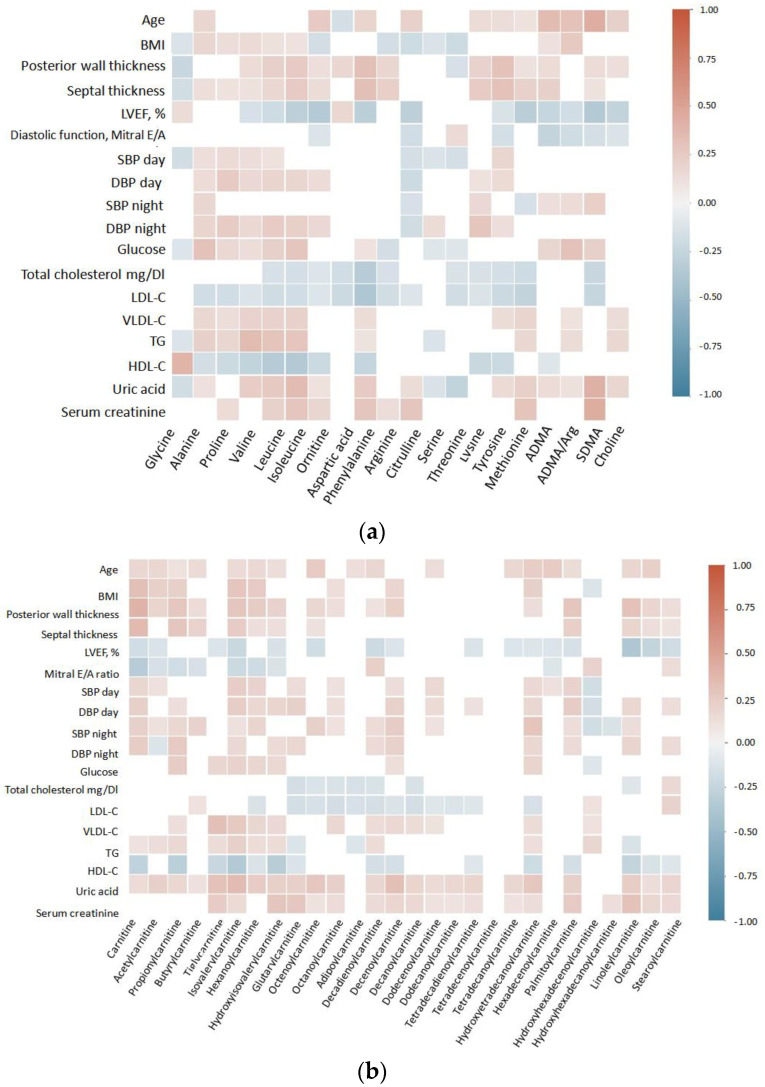

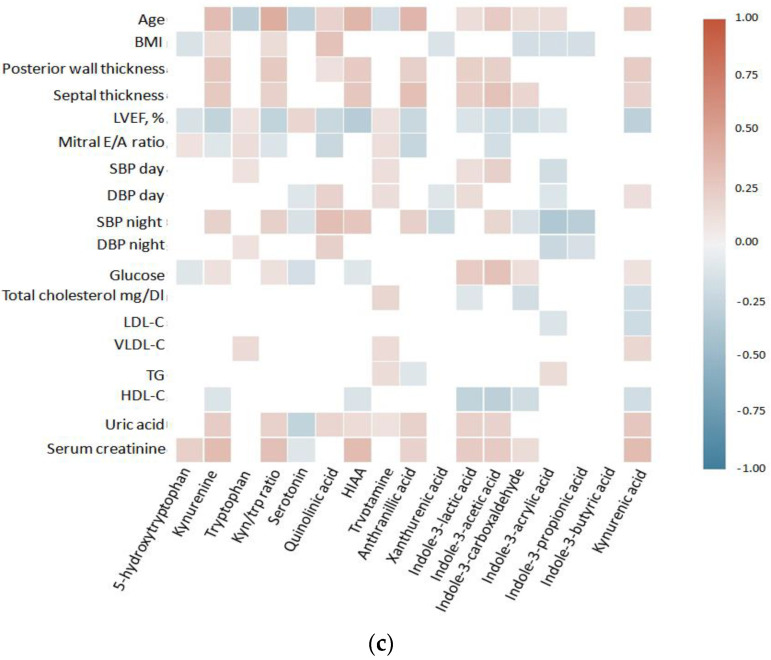
Heatmap correlation matrices between plasma metabolites and cardiometabolic risk factors. (**a**) Amino acids and methylarginines, (**b**) acylcarnitines, and (**c**) tryptophan catabolism metabolites. Each colored square shows a significant Spearman correlation (*p* < 0.05). Red squares depict positive correlations, whereas blue squares are indicative of negative correlations.

To overview associations between the measured metabolomics profiles with clinical cardiological markers, consequent heatmaps were plotted, showing consistent correlation (*p* < 0.05) between cardiometabolic risk factors versus the metabolites (Figure 1a–c). It is important to highlight that LVEF, LDL-C, HDL-C, and uric acid were the most consistent cardiometabolic parameters to be associated with the analyzed metabolites. Additionally, we built correlation heatmaps separately for each group of patients (Supplementary Materials, Figures S1–S3).

### 3.3. Discriminated Biomarkers between the Studied Groups

Discrimination of the metabolites that significantly separated the non-CVD, HTA and CAD groups of the patients was performed using univariate analysis with BH-FDR correction. Moreover, AUC ROC values were calculated to characterize the diagnostic accuracy of each metabolite through the comparison of the non-CVD group versus CVD group (HTA + CAD) and, precisely, the non-CVD group versus the HTA group. Thus, Table 2, Table 3 and Table 4 represent the extracted metabolites related to amino acid, acylcarnitine, and tryptophan metabolism intermediate profiles, respectively. The tables include information on the direction of the alteration along the progression of CVDC, raw *p* value, q value (calculated after the FDR correction), AUC ROC values for non-CVD versus CVD, and, separately, non-CVD versus HTA groups.

Amino acid and methylarginine concentrations were consistently elevated (*p* < 0.05) among the three analyzed groups. The only amino acids that presented a decrease (*p* < 0.05) in concentrations with the progression of the disease were glycine and tryptophan (Table 2). Notably, the Fischer ratio, glycine, ADMA, and ADMA/arginine ratio presented AUCs above 0.7.

All significantly different (*p* < 0.05) acylcarnitines belonging to short-, medium- and long-chain subgroups presented increased concentrations with the progression of the disease (Table 3). Carnitine, short-chain acylcarnitines, hydroxytetradecanoylcarnitine, and palmitoylcarnitine showed AUC values higher than 0.7, reflecting high individual diagnostic power.

Metabolites related to kynurenine and indole metabolic pathways had elevated trends across the progression of the disease, with AUCs higher than 0.7. At the same time, the serotonin pathway showed a declining trend with a corresponding accumulation of HIAA (Table 4). Quantitative levels of the analyzed metabolites are presented in the supplementary material (Table S5).

### 3.4. Application of Machine Learning Modeling for Prediction of CVD

**Figure 2:**
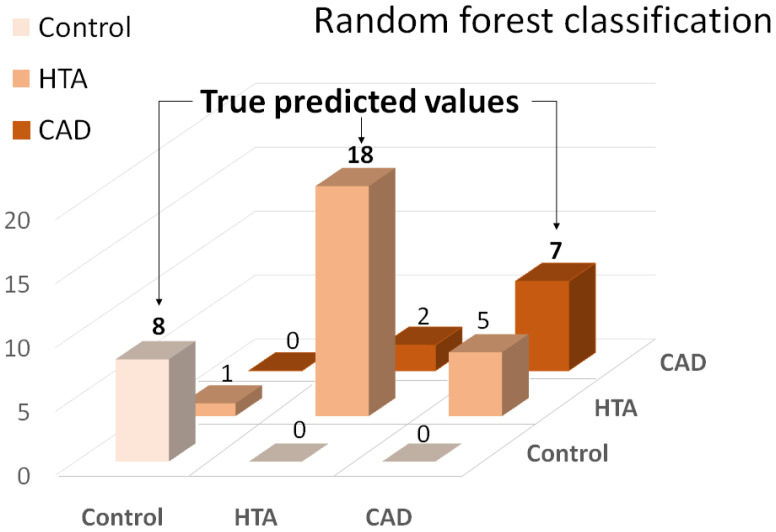
Error matrix of the random forest multiclass classifier.

The development of the predictive CVD ML model was based on the received profiling data. To overview the distribution of the variables, a principal component analysis (PCA) of the three groups (non-CVD group, HTA and CAD) indicated the absence of any object grouping (Supplementary Materials, Figure S4). Therefore, to find the most appropriate ML method, we first performed supervised multiclass classification on the presented three classes (non-CVD group, HTA and CAD) using the four most common multiclass classifiers: random forest, artificial 5 neural networks, gradient boosting and support vector machine. According to the conducted assessment, the random forest classifier showed the highest classification power, with an accuracy equal to 0.8 (Figure 2).

Currently, calculation of the clinical relevance of the ML classification model is typically represented not only through the accuracy assessment but also using additional metrics that may better indicate the efficacy of the model (Table 5). Error matrix plots of the other applied methods are presented in the Supplementary Materials, Figure S4.

Application of the feature importance function resulted in selection of 12 main features, including kynurenine/tryptophan ratio, proline, carnitine, indole-3-acetic acid, hydroxytetradecanoylcarnitine, octenoylcarnitine, ADMA/Arg ratio, propionylcarnitine, ornithine, adipoylcarnitine, butyrylcarnitine, alanine, and 5-hydroxytryptophan (Supplementary Materials, Figure S5). The combination of these metabolites could form a potential panel for stratifying patients with CAD, HTA, and non-CVD individuals.

**Figure 3:**
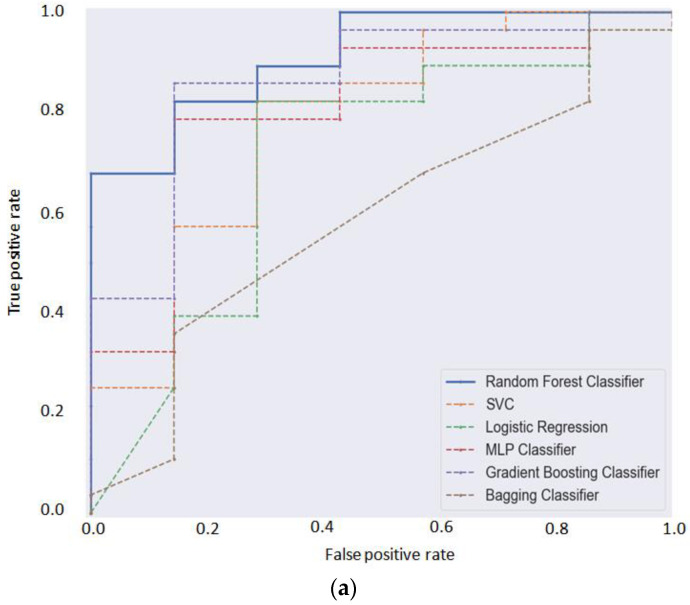

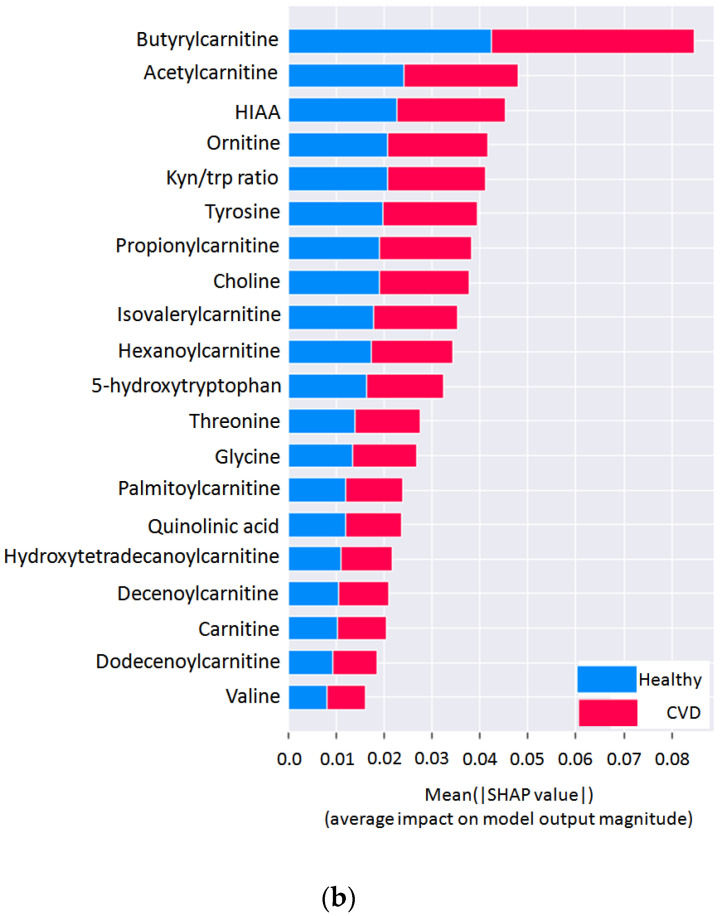
(**a**). Comparison of AUCROCs for the applied algorithms. Random forest showed the highest classification power. (**b**). Selected top variable importance features of the RF model.

Based on the results obtained through multiclass classification, we also applied a binary classification approach among the non-CVD and CVD groups. In this case, six ML algorithms, including logistic regression, random forest classifier, multiple neural network, gradient boosting, support vector classifier, decision tree classifier, and bagging classifier, were applied for the selection of the most appropriate approach. The comparison was performed based on the received quality metrics. The summary metrics of predictive performance for the analyzed models are presented in Table 6. To visualize the effectiveness of the applied classification methods, the appropriate AUCROCs were built (Figure 3a).

According to the results, the random forest algorithm showed the best predictive quality in the classification of patients from the CVD and non-CVD groups (AUCROC = 0.91). Based on the SHAP-based feature importance of the RF model, 10 main features were selected, as presented in Figure 3b.

## 4. Discussion

In the present study, we explored the levels of plasma metabolites measured through targeted metabolomics profiling of patients with CAD, patients with HTA, and non-CVD individuals with the aim of exploring the associations of these metabolites with the progression of the cardiovascular disease continuum.

It is worth noting that through the comparison of the results related to cardiometabolic risk factor patterns of total cholesterol, LDL-C, and HDL-C presented similarities, while VLDL-C presented a descending trend. Presumably, VLDL-C may have more valuable prognostic value than other lipidogram compartments.

**Figure 4:**
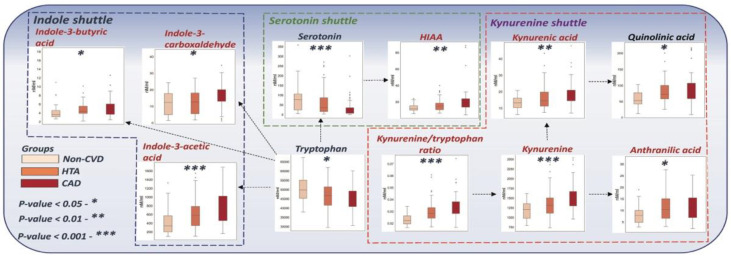
The proposed scheme of the significantly altered metabolites related to tryptophan conversion in blood.

Inflammation plays one of the key roles at all stages of CVD. Likewise, intermediates of the tryptophan catabolism pathway were previously reported to be associated with different stages of CVDC and are therefore predictors of certain acute outcomes, mostly induced through inflammatory processes [30]. Generally, tryptophan is involved in three major metabolic pathways, including the serotonin production pathway, kynurenine pathway, and indole pathway. Figure 4 represents intermediates of tryptophan catabolism that were significantly changed in HTA and CAD patients in comparison to non-CVDs. Consistently, tryptophan, and serotonin plasma concentrations were significantly decreased during CVD progression, suggesting the acceleration of their degradation during inflammation induced by cardiovascular dysregulation. However, increased concentration levels of HIAA indicated its accumulation in blood in CVD patients. The major changes among the considered groups were mainly related to the kynurenine metabolic pathway, which was characterized by significant alterations during early CVDC. Previously, it was found that fatal cardiovascular events have been directly connected with increased conversion of tryptophan to kynurenine [31]. Acceleration of this conversion has a strong association with oxidative stress, inflammation, and immune activation. In this regard, the kynurenine to tryptophan ratio has previously shown a high predictive power for cardiovascular morbidity, including acute coronary events in patients without preexisting coronary artery disease [32,33]. In the present study, we found an elevated trend of the kynurenine/tryptophan ratio across CVD progression, underlining the assumed association of inflammation and early CVD outcomes. Additionally, significantly elevated plasma trends of kynurenic acid, anthranilic acid, and quinolinic acid were also found during the progression of CVDC, which supported the hypothesis of kynurenine pathway activation during cardiovascular disorders. Notably, association of the same elevating trend was previously reported in patients with arterial hypertension compared to non-CVD individuals, where the authors connected these results with hypertensive target organ damage [34]. Overall, we can conclude that the concentration of metabolites related to the kynurenine pathway had elevated trends in CVD groups of patients, reflecting the acceleration of kynurenine pathway activity during the early progression of CVD.

The metabolites that most significantly differentiated the considered groups were related to arginine metabolism intermediates, predominantly comprising the urea cycle pathway. L-arginine is the main substrate of nitric oxide synthase (NOS) for the production of nitric oxide (NO), which is involved in regulatory mechanisms of the cardiovascular system, especially in modulation of vascular tone [35]. In comparison to symmetrical dimethylarginine, asymmetrical dimethylarginine inhibits endogenous nitric oxide synthase and therefore decreases the AOR ratio; thus, increasing ADMA levels suggests the inhibition of nitric oxide production. However, Pope et al. [36] assumed that the associations between arginine metabolites and CVD risks are independent of NO production because the enzymes involved in the metabolism of methylated arginines are affected by inflammation and oxidative stress. Moreover, it should be noted that the concentration of methylated arginine in blood directly correlates with the age of the patients, thereby affecting the interpretation of the received results. The ADMA-to-arginine ratio may also be taken into consideration as a potential biomarker of early-stage CVDC, in which altered levels were previously identified to be associated with the risks of arterial hypertension [37,38].

**Figure 5:**
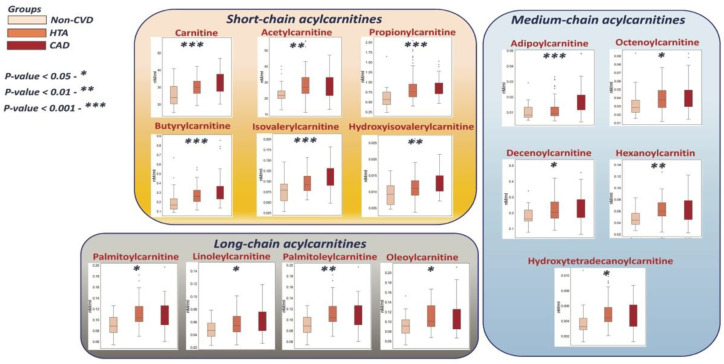
The proposed scheme of the identified significant acylcarnitines.

In addition to glycine, amino acid concentrations have shown a slight increase during CVD progression (Figure 5). In our experiments, significant differences in branched-chain amino acid (BCAA) levels were observed for leucine and isoleucine. Numerous studies have shown strong associations of plasma free amino acids [BCAAs (isoleucine, leucine, and valine), aromatic amino acids, alanine, and proline] with visceral obesity, metabolic disorders, dyslipidemia, hypertension obesity, and type 2 diabetes mellitus [12,39,40]. In particular, increases in BCAA levels in the blood and heart tissues have been associated with the development of cardiovascular and metabolic diseases [41,42,43]. The aromatic amino acids phenylalanine and tyrosine take part in the catabolism of both acetyl-CoA and fumarate. The authors in [44] previously linked an increase in the concentration levels of phenylalanine with CVD using four population cohorts. Interestingly, three amino acids (arginine, ornithine, and citrulline) were inversely proportional to key factors of metabolic syndrome: BMI, LDL-C, total cholesterol, and glucose. Moreover, they were directly connected with a positive factor, VLDL-C, as a positive factor. It should be noted that these amino acids comprise the ornithine cycle responsible for ammonia utilization. Indirectly, the ornithine cycle supports the Cori cycle, which utilizes alanine/lactate and is therefore part of energy metabolism. This finding is supported by the increase in alanine concentration levels in the CVD groups that corresponded to the slowdown of its utilization.

**Figure 6:**
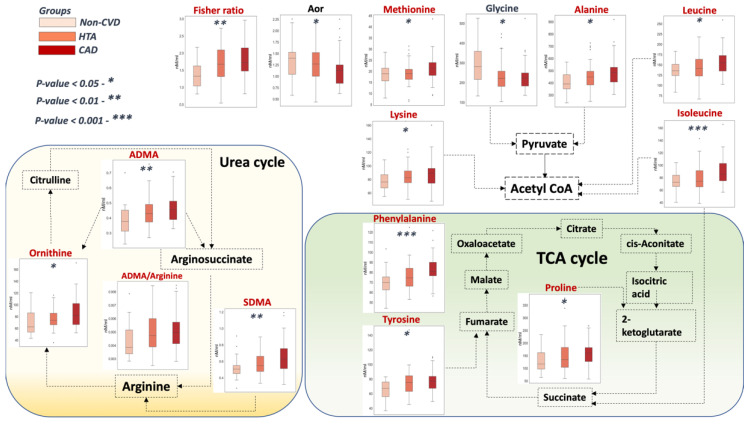
The proposed scheme of the identified significant amino acids associated with the TCA cycle.

As intermediates of fatty acid oxidation, circulating acylcarnitines have been proposed to be connected with the development of CVDs [45,46]. Thus, many previous studies have linked trends of short-chain [46], medium-chain [46,47,48,49], and long-chain [45,46] acylcarnitines with different stages of CVD progression. In our study, significantly different acylcarnitines presented increased concentrations with the progression of CVD, including short-, medium- and long-chain acylcarnitines. It should be noted that not surprisingly, in our study, the patients with CVDs presented a higher BMI than the non-CVD individuals. An accumulation of incompletely oxidized fatty acids in the mitochondria is known as “mitochondrial overload” and, when accompanied by reduced efficiency of glucose disposal, has been reported in obesity [50]. This incomplete fatty acid oxidation is expected to result in the accumulation of mitochondrial-derived acyl-CoA and therefore elevate acylcarnitines [50,51]. The alterations in plasma circulating acylcarnitines may be due to impaired cardiac metabolism connected to obesity. Figure 6 summarizes metabolic pathways and corresponding boxplots of the significantly altered metabolites related to amino acid class.

Data analyses based on univariate analysis are limited and may not fully provide the ability to stratify patients at early stages of CVDC. Application of computational modeling serves for the identification of nonlinear associations among the received metabolomics profiles of the patients. Thus, supervised ML algorithms today serve as superior emerging tools for the prediction of disease stratifications using metabolomics data and are suitable for preliminary postulates of potential biomarkers. ML may model and imitate complex relationships between existing metabolomics changes and predictors that serve to improve early CVD risk stratification. Application of machine learning methods aims to perform an integration assessment of all quantified metabolites resulting in better stratification of patients. While any alteration in concentration level of an individual metabolite could not be considered as the reliable biomarker due to its great concentration biological variability, the presented ML-based approach serves as a more accurate identifier of the disease.

The heterogeneity of cardiological disorders at early stages of CVDC is expected in future investigations, indicating an increased need for the development of multiclass prediction models. However, a relatively small number of approaches have been focused on multiple CVD stage classes using metabolomics profiling data. At the same time, it should be noted that multiclass classification problems are more challenging than binary classification methods. Thus, several classification algorithms applied for binary classification tasks are not always appropriate for binary cases. Most likely, such approaches apply decomposition of the multiclass problem into a number of paired binary subclassification cases, and further combination of these subclassifications results in increased computational time and decreased interpretability of the model decision. Additionally, it should be mentioned that typical for omics studies, the problem of ‘small n, large p’ becomes more crucial in multiclass classification, where subclassification leads to the formation of smaller sample size classes.

According to the study design, we compared three groups of individuals–non-CVD, HTA and CAD groups. Thus, initially we decided to assess the separability of the analyzed groups of patients using a supervised multi-class classification method. There are few reports on the application of multi-class metabolomics approach in cardiology. Despite the controversial quality metrics received for the applied multiclass algorithms, the predictivity accuracy of the random forest classifier equal to 0.8 was relatively acceptable and may be considered as the potential approach for its application in multiclass metabolomics studies.

For the best model fitting, the main hyperparameters were selected using the GridSearchCV approach (scikit-learn package, Python). This approach allowed optimization and provided the best combination of hyperparameters for each of the tested algorithms. In addition, correct evaluation of the ML model performances was reached through the application of a five-fold cross-validation approach for each of the models.

In this regard, to stratify patients with HTA, CAD, and non-CVD individuals, we compared four multiclass classification models, where the random forest algorithm showed the best predictive quality. The error matrix provided clear discrimination of non-CVD patients, while classification of HTA and CAD patients had several misclassification errors. This phenomenon may appear due to non-strict boundaries between the CVD groups of the patients. However, the prediction power of the random forest multiclass model was moderately suitable for the separation of the presented groups of patients (accuracy equal to 0.80). Future studies consisting of larger groups of disease patients may provide more accurate predictions.

Additionally, we performed a binary classification method for separating CVD and non-CVD individuals. The classification results across six classifiers showed the strong possibility of the features differentiating CVD and non-CVD individuals. Thus, the classification performance of the fitted models based on different metrics also indicated that random forest (RF) may provide the best quality and could be chosen as an alternative tool for the identification of CVD patients based on targeted metabolomics profiling. The developed RF models for multivariable and binary classification showed significant quality in separating the patients from the considered groups. The most variable metabolites formed potential metabolomics panels that may be utilized for the preliminary prediction of early stages of CVD based on targeted metabolomics profiling.

The present study was based on the quantification of plasma metabolites in well-discriminated and characterized individuals. However, we acknowledge the lack of follow-up of the patients and statistical analyses performed comparing groups from a cross-sectional study design. It would be ideal to consider stronger study designs, such as prospective cohorts or randomized controlled trials, in the future. Compared to other metabolomics profiling methods, our sample size was not small, but it would be ideal to have a larger sample size to increase the power to detect significant differences.

Nevertheless, our study provides relevant information that can serve translational medicine in cardiovascular risk. Multiple metabolites presented a *p* value > 0.05 to discriminate groups. In comparison to previously published large-scale metabolomics studies, the present experiment is characterized by strict inclusion criteria, resulting in the exclusion of patients with any inflammatory conditions, as well as oncological and endocrine disorders. This study design provides an opportunity to identify metabolomics alterations directly related to the early stages of CVD.

Correlation matrices highlighted cardiometabolic risk factors such as LVEF, LDL-C, HDL-C and uric acid to be significantly correlated with multiple metabolites. One key aspect to consider in the future is to calculate the proper cut points for the concentrations of the metabolites to discriminate among groups. In future studies, based on more robust study designs, it would be interesting to test whether the metabolites found to be discriminated and associated with cardiometabolic risk in our study can be used as biomarkers. This metabolomics profiling contributes to the identification of metabolite changes associated with a higher severity of cardiovascular disorders and shows promise as an early indicator of cardiovascular risk. Moreover, Future studies should also include the assessment of the presented profiles in urine.

In conclusion, the present study provides new findings concerning the significance of plasma metabolites for the prediction of early stages of the CVD continuum. The comparison of different ML algorithms enabled the models based on RF algorithms to provide the best predictive power for the stratification of early CVDC patients based on targeted metabolomics profiling. The presented approach offers prospective starting point for exploring and summarizing the complexity of the interrelated metabolites in cardiology.

## Figures and Tables

**Table 1:** General characteristics of participants.

Variable	Cut Points	Non-CVD Group (n = 27)	Abnormal %	CVD Group (n = 109)				*p* Value		
				HT (n = 61)	Abnormal %	CAD (n = 48)	Abnormal %	Non-CVD vs. HT	Non-CVD vs. CAD	HT vs. CAD
Gender (% w)		50		52		58				
Age, years		48.5 (43–51)		62 (55–69)		65 (59–71)		<0.001	<0.001	<0.05
BMI, kg/m^2^	>30	26.5 (24.7–28.0)	7	31 (29–34)	55	30 (26–34)	44	<0.01	<0.01	NS
Posterior wall thickness, mm (3C)	>10 m	10 (9–10)	0	11 (11–12)	77	11 (10–13)	54	<0.01	<0.01	NS
	>9 w	8.5 (8–10)	14	10 (10–12)	81	10 (10–12)	73	<0.01	<0.01	NS
Septal thickness	>10 m	10 (9–10)	0	11 (10–12)	70	11 (10–13)	57	<0.01	<0.01	NS
	>9 w	8.5 (8–10)	14	10 (9–11)	71	11 (9–12)	50	<0.05	<0.05	NS
LVEF, %	<50	63 (59–66)	0	60 (57–62)	0	57 (50–60)	14	NS	<0.001	<0.001
Diastolic function, Mitral E/A ratio	≤0.8	1.2 (1.1–1.3)	0	0.8 (0.7–1.1)	50	0.7 (0.6–1.0)	40	<0.001	<0.001	NS
SBP day, mmHg	>135	123 (120–130)	3.5	135 (123–144)	31	130 (123–141)	14	<0.01	NS	NS
SBP night, mmHg	>120	103 (99–107)	3.5	121 (111–131)	34	124 (104–134)	12	<0.001	<0.05	NS
DBP day, mmHg	>85	77 (73–81)	10.7	84 (74–90)	33	80 (77–88)	8	NS	NS	NS
DBP night, mmHg	>70	69 (67–75)	25	72 (65–82)	33	71 (69–77)	14	NS	NS	NS
Total cholesterol, mg/dL	>200	203 (175–223)	43	210 (165–231)	55	150 (151–220)	32	NS	NS	NS
LDL-C, mg/dL	>100	101 (85–122)	43	124 (93–142)	56	74 (75–140)	40	NS	NS	NS
VLDL-C, mg/dL	>30	21.4 (14–34)	25	22 (18–33)	25	21 (15–27)	20	NS	NS	NS
HDL-C, mg/dL	<40 m	63 (59–75)	0	57 (48–62)	7	50 (43–71)	7	NS	NS	NS
	<50 w	62 (61–82)	0	55 (48–67)	16	55 (41–64)	23	NS	NS	NS
TG, mg/dL	>150	103 (85–142)	10.7	118 (94–176)	36	104 (77–138)	16	NS	NS	NS
Plasma glucose, mg/dL	100–125	92 (87–96)		99 (904–106)		103 (97–111)		<0.05	<0.001	NS
Serum creatinine, μmol/L	>110 m	94 (93–101)	14	90 (81–96)	13	95.6 (88.0–109.8)	18	NS	NS	NS
	>90 w	88 (85–100)	36	93 (84–107)	48	94 (78–111)	59	NS	NS	NS
Uric acid, mg/dL	>6.99 m	7.2 (6.3–7.4)	43	6.99 (6.24–7.65)	47	6.42 (5.54–7.18)	24	NS	NS	NS
	>5.6 w	5.4 (5.11–5.98)	36	7.1 (5.6–8.0)	65	8.06 (5.95–0.67)	56	NS	<0.01	NS
Smoking (% yes)		3.5		17		24				
GFR, CKD-EPI (mL/min/1.73 m^2^)		71.9 (65.9–77.9)		69.7 (66–73.3)		59.2 (54.4–64.0)		NS	<0.01	<0.01
ACE inhibitor (% yes)		3.5		53		54				
Statins (% yes)		0		41		62				
Angiotensin receptor blockers (% yes)		0		24.2		8.7				
Calcium channel blocker (% yes)		0		24.2		23.9				
Beta-blockers (% yes)		0		33		62				
Diuretics (%yes)		0		33.9		43.5				
Hypoglycemic Medications (% yes)		0		6.5		15.2				

Abbreviations: w = women, m = men, NS = non-significant, GFR = glomerular filtration rate.

**Table 2:** Meaningful metabolites of amino acid profiling selected based on the multiple hypothesis comparison with FDR correction.

Metabolite	Direction	Raw *p*-Value	q-Value	AUC (Non-CVD vs. CVD)	AUC (Non-CVD vs. HTA)
Fischer ratio	Increased	<0.01	<0.01	0.70	0.67
Isoleucine	Increased	<0.01	<0.01	0.62	0.55
Phenylalanine	Increased	<0.00	<0.01	0.66	0.59
ADMA	Increased	0.002	<0.01	0.71	0.67
SDMA	Increased	<0.01	<0.01	0.65	0.60
ADMA/Arginine ratio	Increased	0.011	0.02	0.72	0.71
Ornitine	Increased	0.013	0.02	0.64	0.61
Glycine	Decreased	0.017	0.02	0.68	0.73
Leucine	Increased	0.012	0.020	0.61	0.56
Proline	Increased	0.014	0.022	0.65	0.61
Alanine	Increased	0.019	0.026	0.66	0.63
Lysine	Increased	0.028	0.03	0.64	0.61
Tyrosine	Increased	0.028	0.03	0.66	0.64
Aor	Decreased	0.03	0.03	0.61	0.66
Methionine	Increased	0.04	0.04	0.54	0.51

**Table 3:** Meaningful metabolites of acylcarnitine profiling selected based on the multiple hypothesis comparison with FDR correction.

Metabolite	Direction	Raw *p*-Value	q-Value	AUC (Non-CVD vs. CVD)	AUC (Non-CVD vs. HTA)
Adipoylcarnitine	Increased	<0.00001	<0.001	0.61	0.51
Carnitine	Increased	<0.0001	<0.001	0.75	0.73
Propionylcarnitine	Increased	<0.0001	<0.001	0.77	0.75
Butyrylcarnitine	Increased	<0.0001	<0.001	0.74	0.70
Isovalerylcarnitine	Increased	<0.0001	<0.001	0.73	0.70
Acetylcarnitine	Increased	<0.01	<0.01	0.71	0,73
Hexanoylcarnitine	Increased	<0.01	<0.01	0.72	0.73
Hydroxyisovalerylcarnitine	Increased	<0.01	<0.01	0.64	0.60
Hydroxytetradecanoylcarnitine	Increased	<0.01	<0.01	0.77	0.77
Palmitoylcarnitine	Increased	<0.01	<0.01	0.73	0.75
Octenoylcarnitine	Increased	0.019	0.026	0.67	0.69
Oleoylcarnitine	Increased	0.015	0.027	0.68	0.66
Linoleylcarnitine	Increased	0.021	0.027	0.66	0.65
Hexadecenoylcarnitine	Increased	0.028	0.033	0.68	0.68
Decenoylcarnitine	Increased	0.03	0.03	0.66	0.66

**Table 4:** Meaningful metabolites of tryptophan conversion profiling selected based on the multiple hypothesis comparison with FDR correction.

Metabolite	Direction	Raw *p*-Value	q-Value	AUC (Non-CVD vs. CVD)	AUC (Non-CVD vs. HTA)
Kynurenine/Tryptophan ratio	Increased	<0.00001	<0.0001	0.77	0.72
Serotonin	Decreased	<0.0001	<0.001	0.68	0.61
Indole-3-acetic acid	Increased	<0.0001	<0.001	0.73	0.68
Kynurenine	Decreased	<0.001	<0.001	0.71	0.66
Kynurenic acid	Increased	<0.001	<0.01	0.68	0.61
HIAA	Increased	<0.01	<0.01	0.68	0.62
Quinolinic acid	Increased	0.012	0.020	0.68	0.67
Indole-3-carboxaldehyde	Increased	0.015	0.023	0.59	0.52
Anthranilic acid	Increased	0.032	0.034	0.66	0.65
Tryptophan	Decreased	0.032	0.034	0.65	0.62
Indole-3-butyric acid	Increased	0.049	0.049	0.65	0.64

**Table 5:** Comparison of the main metric characteristics for the applied classification methods.

	Accuracy	F1 Score	Cohen’s Kappa	Log_Loss	AUC Score (One-vs.-All)	Matthews Corr Coef
Random forest classifier	0.80	0.80	0.68	0.67	0.93	0.69
MLP classifier	0.73	0.72	0.55	0.72	0.87	0.57
Gradient classifier	0.66	0.61	0.40	0.68	0.83	0.47
Support vector classifier	0.76	0.75	0.60	0.61	0.88	0.61

**Table 6:** Quality metrics of the applied ML algorithms in the classification of CVD patients and healthy individuals.

Algorithm/Metric	TN	FP	FN	TP	Accuracy	AUC-Score	Precision	Recall	F1
Logistic Regression	3	4	5	23	0.74	0.71	0.84	0.74	0.75
Support vector classifier	0	7	0	28	0.80	0.78	0.80	0.8	0.71
Random Forest Classifier	4	3	0	28	0.91	0.91	0.90	0.91	0.90
MLP Classifier	4	3	4	24	0.8	0.81	0.88	0.8	0.8
Gradient Boosting Classifier	1	6	1	27	0.80	0.86	0.82	0.8	0.75
BaggingClassifier	0	7	0	28	0.8	0.58	0.8	0.8	0.71

## Data Availability

Not applicable.

## Supplementary Materials

The following supporting information can be downloaded at: https://www.mdpi.com/article/10.3390/metabo12121185/s1, Figure S1: Heatmap correlations matrices between plasma metabolites related to carnitine profiling and cardiometabolic risk factors by participant groups: a. Non-CVD individuals; b. Patients with HTA; c. Patients with CAD. Figure S2: Heatmap correlations matrices between plasma metabolites related to amino acid profiling and cardiometabolic risk factors by participant groups: a. Non-CVD individuals; b. Patients with HTA; c. Patients with CAD; Figure S3: Heatmap correlations matrices between plasma metabolites related to tryptophan metabolism intermediates profiling and cardiometabolic risk factors by participant groups: a. Non-CVD individuals; b. Patients with HTA; c. Patients with CAD; Figure S4: Principle component analysis of the three analyzed groups; Figure S5: Error matrixes of models developed using: A-SVC classifier; B–MLP classifier; C–gradient boosting classifier; Figure S6. Plotted feature importance of the multiclass classifier. Table S1: LC-MS/MS parameters used for AA profiling; Table S2: LC-MS/MS parameters used for ADMA and SDMA profiling; Table S3: LC-MS/MS parameters used for AC profiling; Table S4: LC-MS/MS parameters used for tryptophan metabolism profiling; Table S5: Quantitative levels of the analyzed metabolites.
