# Supplementary material for: Target Metabolome Profiling-Based Machine Learning as a Diagnostic Approach for Cardiovascular Diseases in Adults
Source: Metabolites. 2022 Nov 27;12(12):1185. doi: 10.3390/metabo12121185 (PMC9781191; doi:10.3390/metabo12121185)

Validataion procedure of the developed metabolic profiling methods

Metabolite concentrations were calculated according to signal intensity of analytes and appropriate internal standards. Samples from the CAD, HTA and non-CVD groups were analyzed in the same batch. Validation of the developed metabolomic profiling methods was performed in accordance with the US FDA and EMA guidelines for bioanalytical method validation (EMA, 2019; USFDA, 2018). Validation procedure included assessment of the following parameters: selectivity, linearity, precision and accuracy, recovery, matrix effect, and stability. Isotopically labeled internal standards (ISTD) solution were applied as Quality control samples to monitor data enrichment and instrumental performance. Weighted (1/x) linear regression analysis included calculation of the R2 value, slope, and intercept and was used to construct calibration curves. To assess he inter- and intra- assay precision and accuracy there were analyzed QC samples in six replicates through three analytical runs. Stability tests were performed in QC samples at low QC and high QC levels using working solutions of the analytes placed at room temperature (21 ± 3 °C), using biological samples placed in the autosampler at 10 ± 0.5 °C for 24 hours; using biological samples at 35 ± 1 °C during 20 days. Recovery and matrix effects were assessed in low- and high-level QC samples.

Table S1. LC-MS/MS parameters used for AA profiling

Name	Retention time	Precursor ion	Product ion	Cone	CE	Internal Standard
Gly	1.21	76.0	30.0	17	10	13C215N-Gly
13C215N-Gly	1.21	79.0	32.0	17	10	
Ala	1.24	90	44	18	10	D4-Ala
D4-Ala	1.24	94	48	18	10	
Pro	1.37	116.1	70.1	11	13	D4-Ala
Orn	1.08	133.1	70.1	18	20	D6-Orn
D6-Orn	1.08	139.1	76.1	18	20	
Leu	2.94	132.1	86.1	17	10	D3-Leu
D3-Leu	2.91	135.1	89.1	17	10	
ILe	2.68	132.1	86.1	17	10	D3-Leu
Val	1.5	118.1	72.1	19	13	D8-Val
D8-Val	1.5	126.1	80.1	19	13	
Asp	1.26	134	116.1	20	13	D3-Asp
D3-Asp	1.26	137	119.1	20	13	
Glu	1.29	148.1	130	20	13	D5-Glu
D5-Glu	1.29	153	135	20	13	
Met	1.93	150.1	133	18	10	D3-Met
D3-Met	1.93	153.1	136	18	10	
Phe	3.74	166.1	120.1	20	12	D5-Phe
D5-Phe	3.74	171	125	20	12	
Arg	1.11	175.1	70.1	22	21	D7-Arg
D7-Arg	1.11	182	77	22	21	
Cit	1.28	176.1	113.1	16	16	D2-Cit
D2-Cit	1.28	178.1	115.1	16	16	
Tyr	2.47	182.1	136.1	20	13	D4-Tyr
D4-Tyr	2.47	186	140	20	13	
Ser	1.22	106	60	20	13	D4-Ala
Thr	1.28	120	56	20	13	D5-Phe
Lys	1.08	147	84	20	13	D4-Ala
Trp	4.23	205	188	20	13	D5-Phe
His	1.10	156	110	20	13	D4-Ala

Table S2. LC-MS/MS parameters used for ADMA and SDMA profiling

Name	Retention time	Precursor ion	Product ion	Cone	CE	Internal Standard
Arg	2.04	175.1	70.1	22	21	D7-Arg
D7-Arg	2.04	76.0	30.0	22	21	
ADMA	3.76	203.2	46.1	22	21	D7-Arg
SDMA	3.53	203.2	172.1	22	21	D7-Arg
Choline	2.85	60.1	104.1	22	21	D7-Arg

Table S3. LC-MS/MS parameters used for AC profiling

Analyte	Abbreviation	Precursor ion	Product ion	CE
Carnitine	C0	162	103	17
Carnitine-D9	C0-ISTD	171	103	17
Acetylcarnitine	C2	204	85	18
Acetylcarnitine-D3	C2-ISTD	207	85	18
Propionylcarnitine	C3	218	85	19
Propionylcarnitine -D3	C3-ISTD	221	85	19
Butyrylcarnitine	C4	232	85	20
Butyrylcarnitine-D3	C4-ISTD	235	85	20
Valerylcarnitine	C5	246	85	20
Valerylcarnitine-D9	C5-ISTD	255	85	20
Tiglylcarnitine	C5:1	244	85	20
Glutarylcarnitine	C5-DC	276	85	20
Hexanoylcarnitine	C6	260	85	22
Hexanoylcarnitine-D3	C6-ISTD	263	85	22
Octanoylcarnitine	C8	288	85	24
Octanoylcarnitine -D3	C8-ISTD	291	85	24
Octenoylcarnitine	C8:1	286	85	24
Decanoylcarnitine	C10	316	85	26
Decanoylcarnitine -D3	C10-ISTD	319	85	26
Decenoylcarnitine	C10:1	314	85	26
Decadienylcarnitine	C10:2	312	85	26
Dodecanoylcarnitine	C12	344	85	27
Dodecanoylcarnitine -D3	C12-ISTD	347	85	27
Dodecenoylcarnitine	C12:1	342	85	27
Tetradecadienylcarnitine	C14:2	368	85	27
Hexadecanoylcarnitine	C16	400	85	28
Hexadecanoylcarnitine -D3	C16-ISTD	403	85	28
Octadecanoylcarnitine	C18	428	85	28
Octadecanoylcarnitine -D3	C18-ISTD	431	85	28
Octadecenoylcarnitine	C18:1	426	85	26
Octadecadienylcarnitine	C18:2	424	85	20
Hydroxyvalerylcarnitine	C5-OH	262	85	26
Hydroxyhexadecenoylcarnitine	C16:1-OH	398	85	26
Hydroxyoctadecenoylcarnitine	C18:1-OH	442	85	28

Table S4. LC-MS/MS parameters used for tryptophan metabolism profiling

Analyte	RT, min	Registered ions		Fragmentor	CE
		Precursor ion	Product ion		
1-Methyltryptophan	4.2	219	146	90	20
3-Aminoisobutyric acid	2.3	104	57	90	15
3-Hydroxyanthranilic acid	5.1	154	136	90	10
3-Hydroxykynurenine	3.9	225	162; 110	90	22; 17
3-Hydroxyanthranilic-d3 acid	5.0	157	139.1	90	10
3-Hydroxykynurenine 13C2, 15N	3.9	228.1	110.1	90	17
4-Aminobutyric acid	2.0	104	87; 69	90	10; 15
4-Aminobutyric-d6 acid	2.0	110.1	93.2; 73.2	90	10; 15
5-Hydroxytryptophan	5.4	221	204; 162	90	10; 20
5-Hydroxytryptophol	5.9	178	160	100	15
5-Methoxytryptamine	3.0	191	174	90	10
6-Hydroxymelatonin	6.5	249	190	110	15
Acetylcholine	5.2	146	87; 60; 43	90	15; 10
Anthranilic acid	6.6	138.1	120.1; 92	95	10; 20
Asparagine	1.1	133	87; 74	100	20
Aspartic acid	1.1	134	87; 74	100	10; 15
Biopterin	2.5	238.1	194; 178	90	20
Biopterin-d3	2.5	241	194	90	20
Choline	2.2	104	60	90	20
Citrulline	1.3	176	113; 70	90	20; 30
Cortisol	7.3	363	327; 121	120	10; 20
Dopamine	4.8	154	119; 91	90	10
Dopamine-d4	4.9	158.1	123.1; 95.1	90	10; 30
Epinephrine	3.6	184	166; 151	90	5; 25
Glutamic acid	1.3	148	84; 56	100	15; 30
Glutamine	1.2	147	130; 84	100	10; 15
HIAA	6.1	192.1	146.1	100	20
HIAA-d5	6.2	197	150.1	100	20
Homovanillic acid	6.3	181.1	137; 122	70	4; 16
Indole-3-acetic acid	7.2	176.1	130.1	95	20
Indole-3-acetic-d4 acid	7.2	180.1	133.1	95	20
Indole-3-acryllic acid	7.3	188.1	170; 115	100	20; 25
Indole-3-butyric acid	7.7	204.2	144.2; 130.1	100	25
Indole-3-butyric-d4 acid	7.8	208	132	100	25
Indole-3-carboxaldehyde	7.2	146.1	118.1; 91.1	95	15; 30
Indole-3-carboxaldehyde-13C8	7.3	154.1	126.1; 98.1	95	30
Indole-3-lactic acid	6.8	206.1	118.1	95	22
Indole-3-lactic-d5 acid	6.9	211	122	95	22
Indole-3-propionic acid	7.5	190.1	130.1	95	20
Indole-3-propionic-d2 acid	7.6	192.1	130.1	95	20
Kynurenic acid	7.1	190.1	144.1	95	20
Kynurenic-d5 acid	6.7	195.1	149.1	95	30
Kynurenine	5.9	209.1	146; 94	95	30; 10
Kynurenine-d4	5.9	213	98.1	95	10
L-DOPA	3.0	198	181; 152	100	10
L-Tryptophan	7.3	203	142; 116	100	15
L-Tryptophan-d5	6.7	208.3	164.3; 120.1	100	15
Melatonin	7.2	233	174	100	15
Melatonin-d4	7.2	237	178	120	10
Metanephrine	6.0	198	165; 148	90	20; 25
N-acetyl-5-hydroxytryptamine	6.2	219	160	100	15
Neopterin	1.6	254.1	206.1; 190.1	100	20
Neopterin-13C5	1.7	259	210	120	10
N-methylserotonin	8.6	191	160	90	12
Norepinephrine	2.5	152	107	110	20
Normetanephrine	4.6	166	134	100	15
Normetanephrine-d3	4.7	187.2	137.1	100	15
Phenylalanine	5.8	166	120	100	15

Quinolinic acid	2.7	168.1	106.1; 78.1	95	15; 30
Quinolinic acid-d3	1.9	171.1	109.1; 81.1	95	15; 30
Serotonin	7.3	177; 160	160; 132; 105	90; 120	7; 20; 30
Serotonin-d4	7.5	181.1	164; 136	90; 120	7; 20
Tryptamine	8.0	161.1	144.1; 115.1	95	10; 30
Tryptophol	7.2	162.1	144.2	100	13
Tyrosine	3.7	182	165; 136	90	5; 10
Vanilylmandelic acid	3.5	197	138; 137	100	10; 24
Xanthurenic acid	6.7	206.1	160; 132.1	95	20; 30
Xanthurenic-d4 acid	6.8	210.1	164.1; 136.1	95	20; 30

Table S5. Concentration (median and interquartile range)

Metabolite	Non-CVD		HTA		CAD	
	Median	Quartile range	Median	Quartile range	Median	Quartile range
Amino acids, uM						
GABR	0.85	0.70 - 0.94	0.78	0.63 - 0.95	0.72	0.58 - 0.84
Aor	1.39	1.04 - 1.53	1.27	1.00 - 1.52	1.13	0.85 - 1.25
Fischer ratio	1.33	1.05 - 1.63	1.68	1.32 - 2.09	1.72	1.48 - 2.15
Glycine	281	216.3 - 359.0	222.6	181.5 - 258.4	224.3	183.4 - 249.3
Alanine	392	352.1 - 467.6	449.4	383.9 - 495.1	487.1	407.9 - 528.1
Proline	117	97.2 - 162.1	134.2	104.2 - 182.9	160.9	127.3 - 182.3
Valine	240	226.1 - 265.2	257.8	225.7 - 295.1	262.0	236.5 - 308.6
Leucine	135.8	124.2 - 151.4	141.3	122.9 - 163.6	155.0	135.0 - 172.8
Isoleucine	72.86	65.97 - 83.98	74.37	65.40 - 91.39	85.82	75.10 - 103.02
Ornithine	62.57	53.77 - 86.33	73.81	66.31 - 85.81	81.95	66.62 - 102.28
Aspartic acid	5.62	4.38 - 7.35	6.20	5.02 - 7.68	6.04	4.65 - 7.22
Phenylalanine	69.83	62.65 - 75.48	74.60	66.23 - 84.16	80.91	76.66 - 90.09
Arginine	86.38	77.00 - 100.25	94.87	79.82 - 105.46	91.67	80.76 - 101.55
Citrulline	40.43	31.44 - 44.27	40.09	34.55 - 48.77	44.22	37.05 - 50.14
Serine	99.69	83.86 - 120.16	91.90	79.87 - 108.06	94.90	83.66 - 106.29
Threonine	110.37	97.64 - 114.29	98.71	84.97 - 108.96	100.48	87.24 - 115.22
Lysine	76.34	67.49 - 87.11	82.61	75.31 - 92.66	89.37	74.54 - 96.41
Tyrosine	67.37	55.98 - 76.09	75.19	63.20 - 84.61	77.13	67.42 - 83.82
Methionine	18.95	15.703 - 21.39	18.95	16.38 - 20.91	21.33	18.18 - 23.95
ADMA	0.38	0.31 - 0.45	0.43	0.37 - 0.49	0.46	0.39 - 0.51

ADMA/Arg	0.0038	0.0033 - 0.005	0.00	0.00 - 0.01	0.00	0.00 - 0.01
SDMA	0.506	0.451 - 0.550	0.55	0.48 - 0.66	0.63	0.51 - 0.76
Choline	12.25	11.24 - 14.08	12.91	11.40 - 17.54	13.89	11.73 - 17.03
Carnitine	23.98	20.43 - 30.56	29.93	26.49 - 33.62	33.39	27.70 - 37.63
Acylcarnitines, uM						
Acetylcarnitine	21.84	19.91 - 24.69	26.99	23.10 - 33.17	27.23	21.82 - 33.18
Propionylcarnitine	0.57	0.44 - 0.75	0.76	0.63 - 0.95	0.84	0.71 - 0.98
Butyrylcarnitine	0.167	0.12 - 0.23	0.26	0.20 - 0.33	0.28	0.23 - 0.37
Tiglylcarnitine	0.009	0.007 - 0.011	0.01	0.01 - 0.01	0.01	0.01 - 0.01
Isovalerylcarnitine	0.079	0.054 - 0.094	0.09	0.08 - 0.11	0.11	0.09 - 0.13
Hexanoylcarnitine	0.044	0.037 - 0.056	0.06	0.05 - 0.07	0.06	0.05 - 0.08
Hydroxyisovalerylcarnitine	0.009	0.006 - 0.011	0.01	0.01 - 0.01	0.01	0.01 - 0.01
Glutarylcarnitine	0.131	0.108 - 0.172	0.13	0.11 - 0.17	0.15	0.13 - 0.18
Octenoylcarnitine	0.028	0.023 - 0.037	0.04	0.03 - 0.05	0.03	0.03 - 0.05
Octanoylcarnitine	0.152	0.103 - 0.185	0.16	0.13 - 0.21	0.18	0.13 - 0.23
Adipoylcarnitine	0.008	0.007 - 0.013	0.01	0.01 - 0.01	0.02	0.01 - 0.02
Decadienoylcarnitine	0.017	0.014 - 0.020	0.02	0.02 - 0.02	0.02	0.02 - 0.02
Decenoylcarnitine	0.163	0.149 - 0.219	0.21	0.17 - 0.27	0.23	0.17 - 0.29
Decanoylcarnitine	0.221	0.159 - 0.303	0.24	0.19 - 0.33	0.28	0.18 - 0.36
Dodecenoylcarnitine	0.062	0.0451 - 0.071	0.07	0.06 - 0.09	0.08	0.05 - 0.10
Dodecanoylcarnitine	0.062	0.044 - 0.080	0.07	0.06 - 0.09	0.07	0.05 - 0.10
Tetradecadienoylcarnitine	0.032	0.026 - 0.038	0.04	0.03 - 0.05	0.04	0.03 - 0.05
Tetradecenoylcarnitine	0.058	0.040 - 0.070	0.06	0.05 - 0.09	0.07	0.04 - 0.09
Tetradecanoylcarnitine	0.022	0.017 - 0.027	0.03	0.02 - 0.03	0.03	0.02 - 0.03
Hydroxytetradecanoylcarnitine	0.003	0.002 - 0.004	0.00	0.00 - 0.01	0.00	0.00 - 0.01
Hexadecenoylcarnitine	0.022	0.018 - 0.027	0.03	0.02 - 0.03	0.03	0.02 - 0.04
Palmitoylcarnitine	0.089	0.077 - 0.105	0.10	0.10 - 0.12	0.11	0.09 - 0.13
Hydroxyhexadecenoylcarnitine	0.045	0.038 - 0.051	0.04	0.03 - 0.05	0.04	0.04 - 0.05
Hydroxyhexadecanoylcarnitine	0.002	0.0011 - 0.0018	0.00	0.00 - 0.00	0.00	0.00 - 0.00
Linoleylcarnitine	0.047	0.037 - 0.058	0.05	0.04 - 0.07	0.06	0.05 - 0.08
Oleoylcarnitine	0.091	0.076 - 0.104	0.10	0.09 - 0.13	0.11	0.08 - 0.13

Stearoylcarnitine	0.035	0.031 - 0.040	0.04	0.03 - 0.04	0.04	0.03 - 0.04
Tryptophan metabolism intermediates, nM						
5-Hydroxytryptophan	0.021	0.015 - 0.031	0.02	0.01 - 0.02	0.02	0.01 - 0.02
Kynurenine	1204	981 - 1355	1312	1113 - 1504	1492	1301 - 1668
Tryptophan	49823	45366 - 55123	46827	41583 - 51495	45178	40862 - 49211
Kyn/Trp	22.45	19.49 - 26.41	28.45	24.34 - 33.19	32.60	28.27 - 38.09
Serotonin	7.73	2.51 - 10.63	3.66	1.58 - 8.71	1.37	0.73 - 3.54
Quinolinic acid	53.31	42.69 - 77.86	72.16	58.46 - 100.57	74.80	59.29 - 107.27
HIAA	0.012	0.010 - 0.015	0.01	0.01 - 0.02	0.02	0.01 - 0.02
Tryptamine	0.098	0.076 - 0.129	0.11	0.08 - 0.14	0.10	0.06 - 0.13
Anthranillic acid	7.889	4.845 - 10.056	10.36	6.65 - 15.09	11.03	6.98 - 15.43
Xanthurenic acid	0.016	0.010 - 0.032	0.02	0.01 - 0.03	0.02	0.01 - 0.03
Indole-3-lactic acid	359	254 - 459	357.93	301.54 - 503.12	411.37	315.25 - 505.56
Indole-3-acetic acid	341	200 - 572	581.39	346.12 - 786.62	728.28	462.21 - 1022.32
Indole-3-carboxaldehyde	12.37	4.92 - 17.74	12.77	5.53 - 18.09	16.31	13.13 - 20.10
Indole-3-acrylic acid	11.08	8.48 - 21.84	14.31	9.68 - 20.90	19.24	12.64 - 24.37
Indole-3-propionic acid	163.01	117.96 - 246.21	201.73	123.93 - 349.38	317.03	156.86 - 415.15
Indole-3-butyric acid	3.64	3.12 - 4.56	4.40	3.93 - 5.55	4.80	3.69 - 6.05
Kynurenic acid	13.36	10.34 - 16.21	14.47	11.27 - 19.83	18.06	14.52 - 20.99

Figure S1. Heatmap correlations matrices between plasma metabolites related to carnitine profiling and cardiometabolic risk factors by participant groups: a. Non-CVD (healthy) individuals; b. Patients with HTA; c. Patients with CAD.

A.

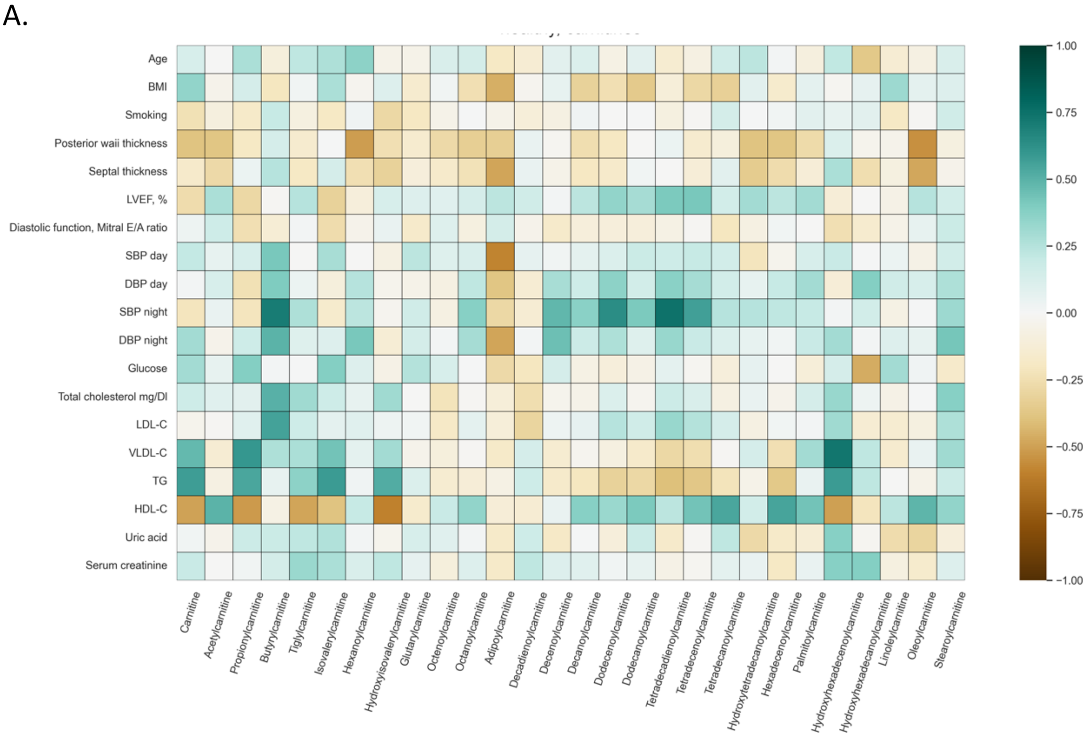

B

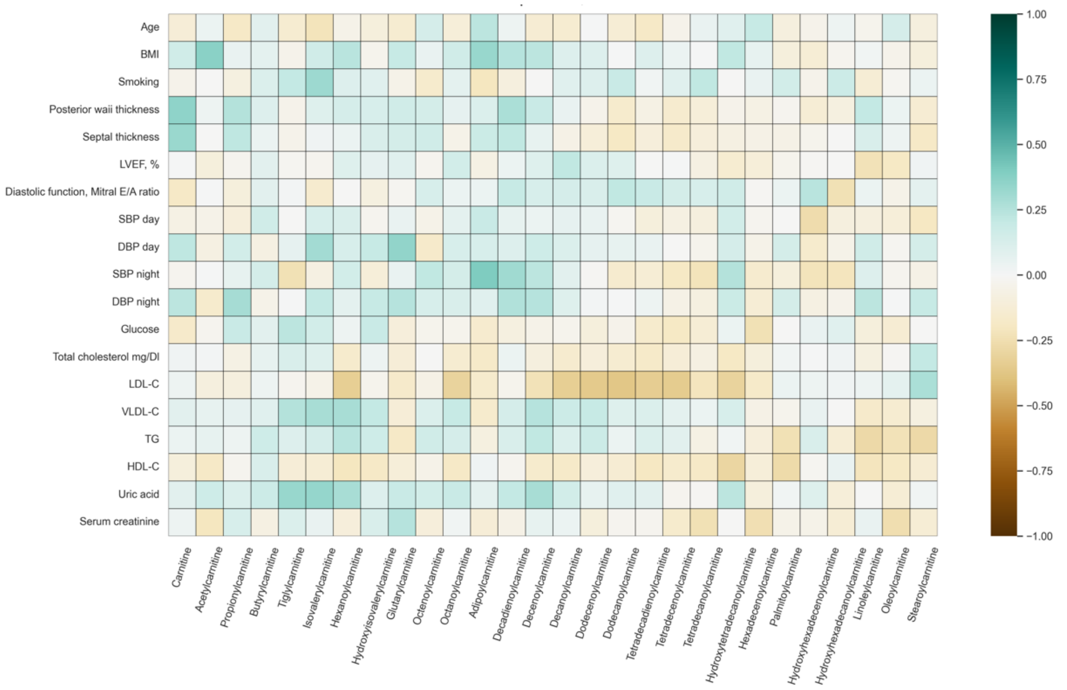

.

C

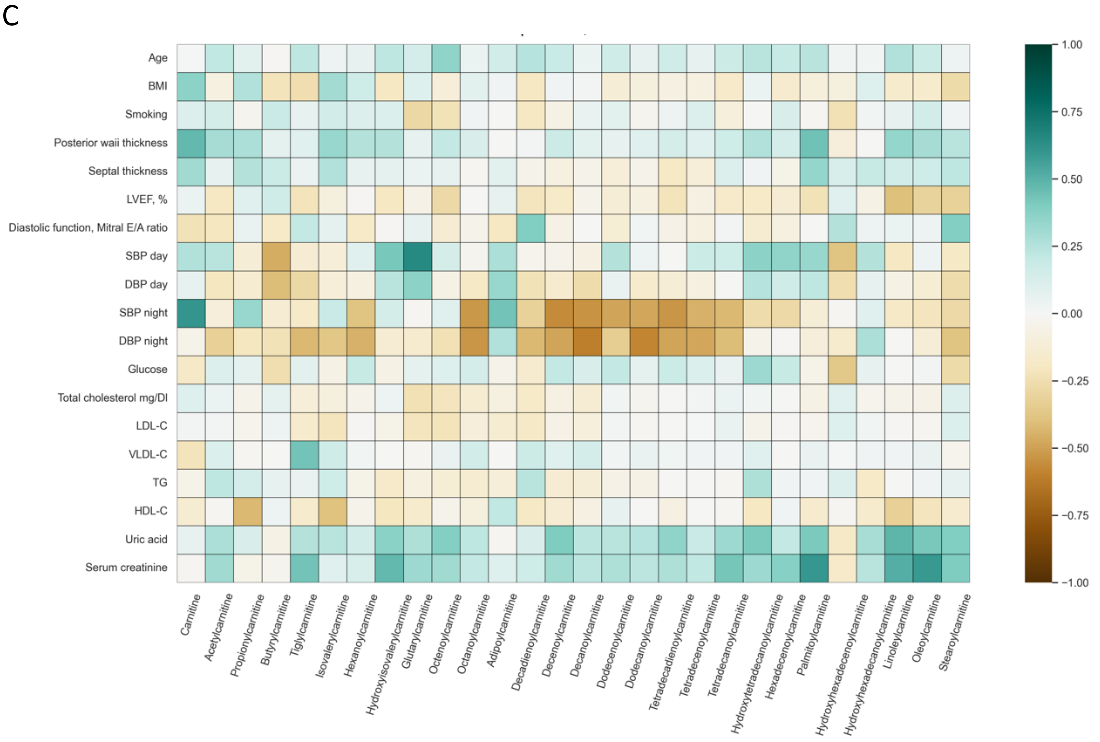

Figure S2. Heatmap correlations matrices between plasma metabolites related to amino acid profiling and cardiometabolic risk factors by participant groups: a. Non-CVD individuals; b. Patients with HTA; c. Patients with CAD.

A

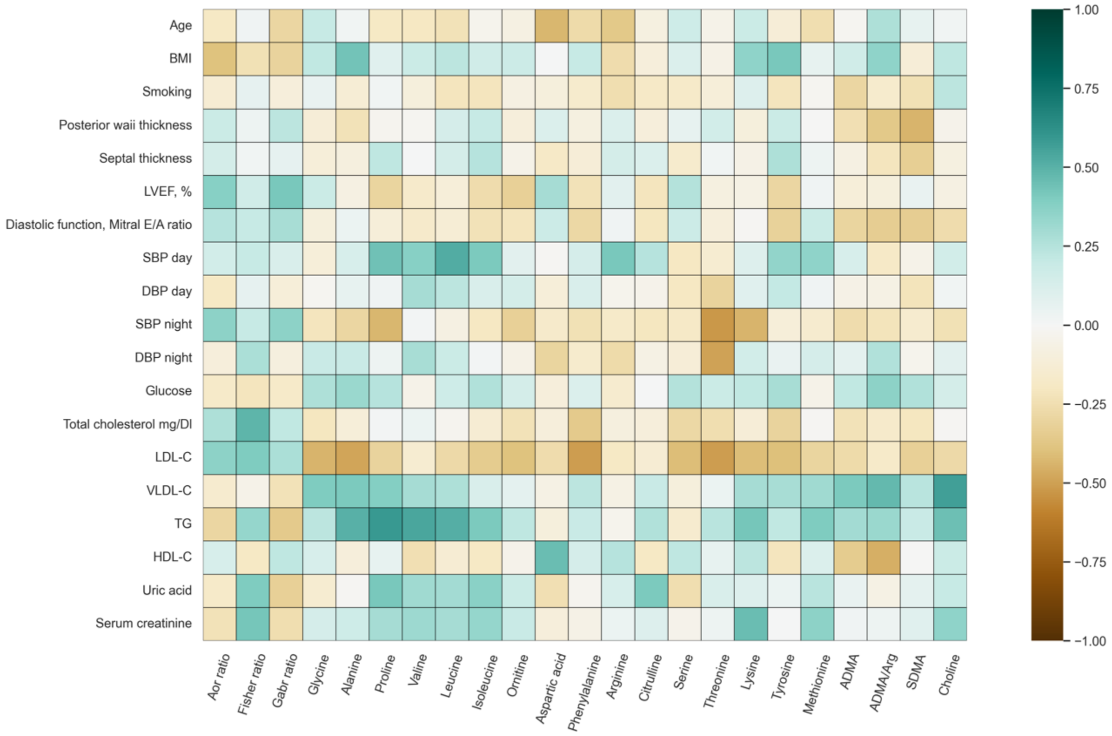

B

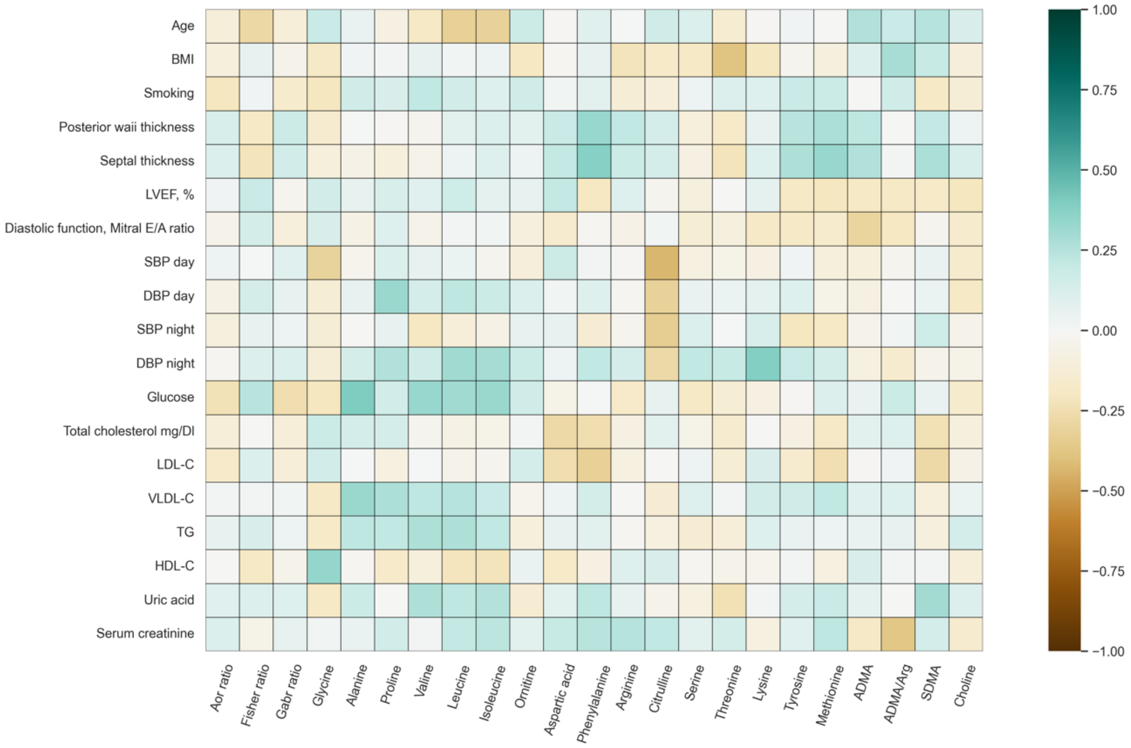
Age
BMI
Smoking
Posterior waii thickness
Septal thickness
LVEF, %
Diastolic function, Mitral E/A ratio
SBP day
DBP day
SBP night
DBP night
Glucose
Total cholesterol mg/Dl
LDL-C
VLDL-C
TG
HDL-C
Uric acid
Serum creatinine
Aor ratio
Fisher ratio
Gabr ratio
Glycine
Alanine
Proline
Valine
Leucine
Isoleucine
Ornitine
Aspartic acid
Phenylalanine
Arginine
Citrulline
Serine
Threonine
Lysine
Tyrosine
Methionine
ADMA
ADMA/Arg
SDMA
Choline
1.00
0.75
0.50
0.25
0.00
−0.25
−0.50
−0.75
−1.00

C

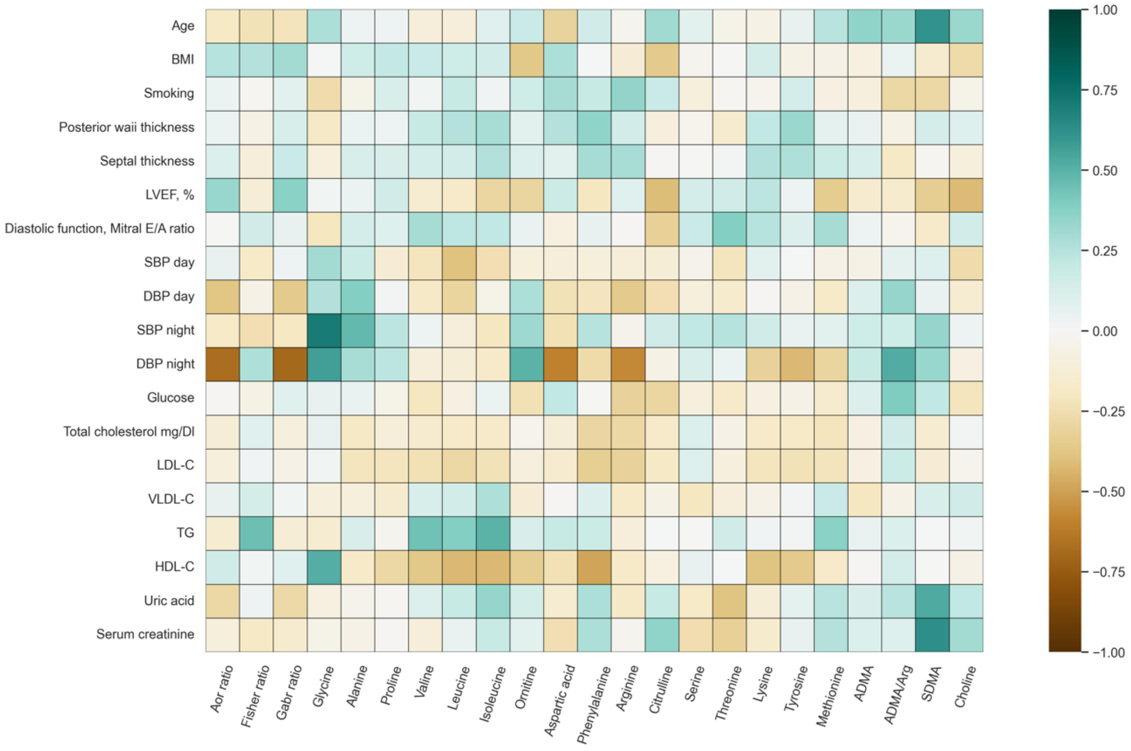
Age
BMI
Smoking
Posterior waii thickness
Septal thickness
LVEF, %
Diastolic function, Mitral E/A ratio
SBP day
DBP day
SBP night
DBP night
Glucose
Total cholesterol mg/Dl
LDL-C
VLDL-C
TG
HDL-C
Uric acid
Serum creatinine
Aor ratio
Fisher ratio
Gabr ratio
Glycine
Alanine
Proline
Valine
Leucine
Isoleucine
Ornitine
Aspartic acid
Phenylalanine
Arginine
Citrulline
Serine
Threonine
Lysine
Tyrosine
Methionine
ADMA
ADMA/Arg
SDMA
Choline
1.00
0.75
0.50
0.25
0.00
−0.25
−0.50
−0.75
−1.00

Figure S3. Heatmap correlations matrices between plasma metabolites related to tryptophan metabolism intermediates profiling and cardiometabolic risk factors by participant groups: a. Healthy individuals; b. Patients with HTA; c. Patients with CAD.

A.

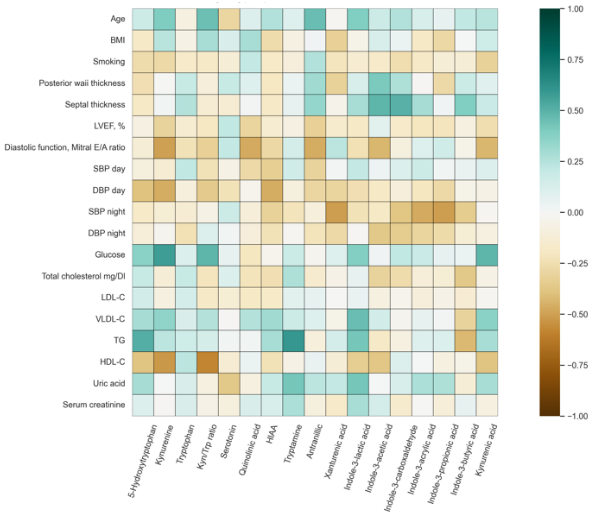

B.

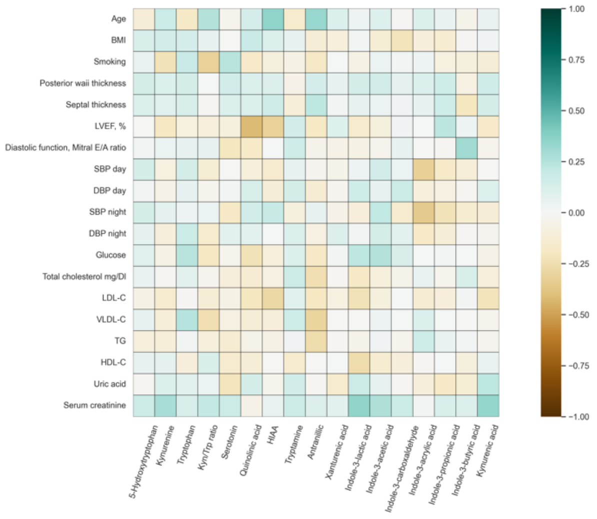

C.

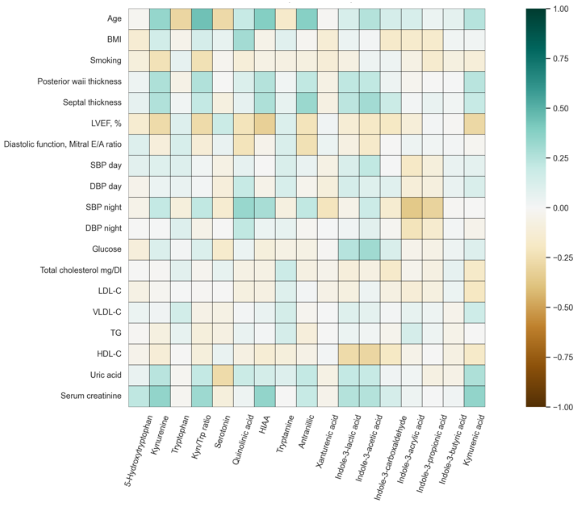

Figure S4. Principle component analysis of the three analyzed groups

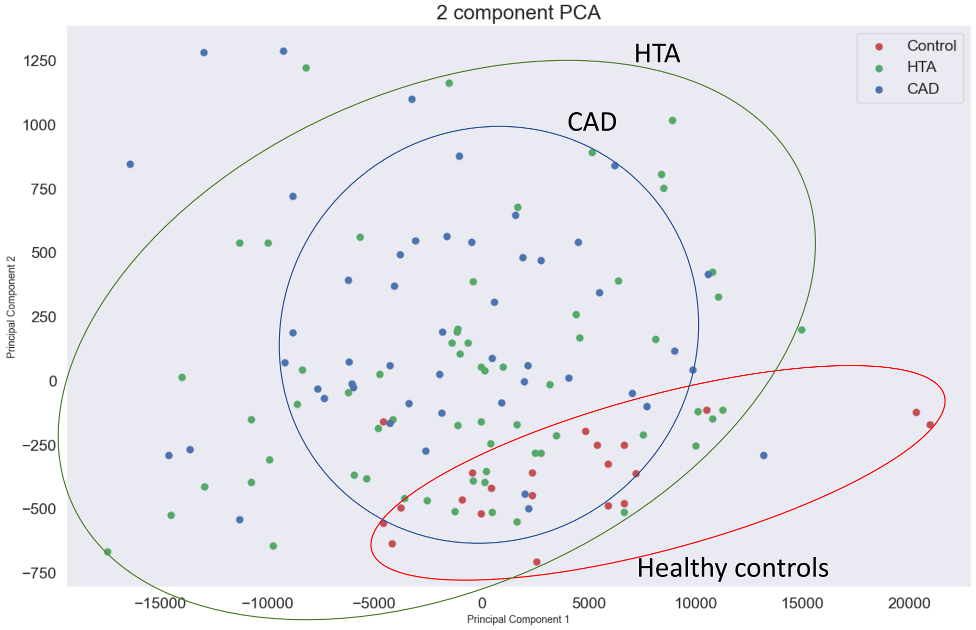

Figure S5. Error matixes of models developed using: A - SVC classifier; B – MLP classifier; C – Gradient boosting classifier.

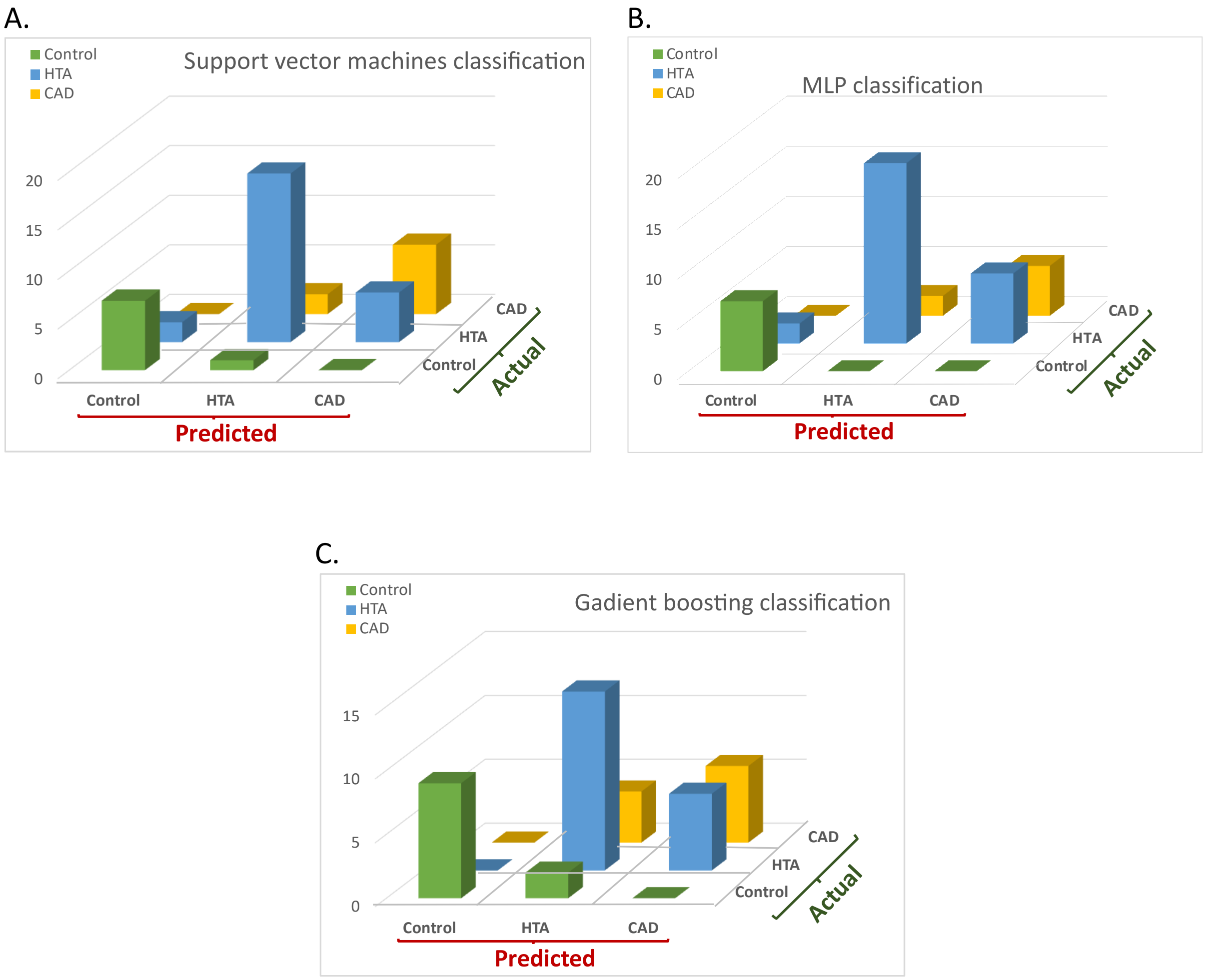

Figure S6. Plotted feature importance of the multiclass classifier

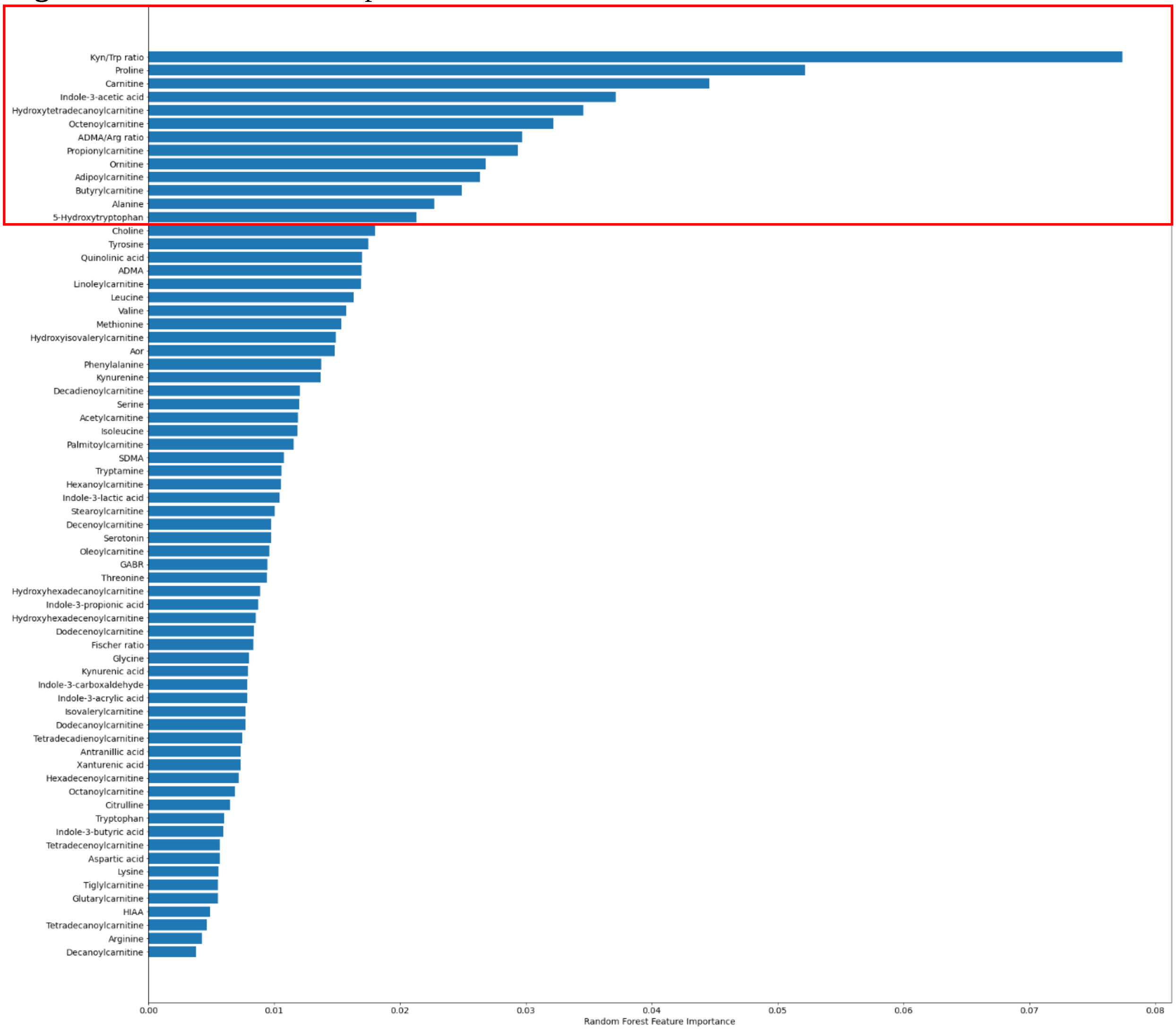

Supplement: Supplementary file 1 [file metabolites-12-01185-s001.zip › metabolites-2022001-supplementary.pdf]
